# Triboelectric Effect Enabled Self-Powered, Point-of-Care Diagnostics: Opportunities for Developing ASSURED and REASSURED Devices

**DOI:** 10.3390/mi12030337

**Published:** 2021-03-22

**Authors:** Navneet Soin, Sam J. Fishlock, Colin Kelsey, Suzanne Smith

**Affiliations:** 1 School of Engineering, Ulster University, Belfast BT37 0QB, Northern Ireland, UK; s.fishlock@ulster.ac.uk (S.J.F.); c.kelsey@ulster.ac.uk (C.K.); 2 Department of Electrical, Electronic and Computer Engineering, University of Pretoria, Pretoria 0028, South Africa

**Keywords:** ASSURED devices, REASSURED devices, point-of-care devices, energy harvesting, self-powered, triboelectric nanogenerators

## Abstract

The use of rapid point-of-care (PoC) diagnostics in conjunction with physiological signal monitoring has seen tremendous progress in their availability and uptake, particularly in low- and middle-income countries (LMICs). However, to truly overcome infrastructural and resource constraints, there is an urgent need for self-powered devices which can enable on-demand and/or continuous monitoring of patients. The past decade has seen the rapid rise of triboelectric nanogenerators (TENGs) as the choice for high-efficiency energy harvesting for developing self-powered systems as well as for use as sensors. This review provides an overview of the current state of the art of such wearable sensors and end-to-end solutions for physiological and biomarker monitoring. We further discuss the current constraints and bottlenecks of these devices and systems and provide an outlook on the development of TENG-enabled PoC/monitoring devices that could eventually meet criteria formulated specifically for use in LMICs.

## 1. Introduction

The realization of effective point-of-care (PoC) diagnostics, particularly for low- and middle-income countries (LMICs), has seen tremendous advances in recent decades. However, various challenges are yet to be overcome for these solutions to be truly successful, including the need for self-powered devices. This review provides an overview and outlook on self-powered advances in healthcare and PoC devices towards solutions that meet criteria formulated specifically for use in LMICs.

Accurate, rapid diagnosis is a critical component of healthcare that drives the prognosis, treatment, and clinical outcome for patients [1]. The past decades have seen diagnostics being primarily driven by a centralized-laboratory dominant system, enabling automated and high-volume multiplexed analysis at a relatively low cost [1,2,3]. More recently, this healthcare model has shifted to a more localized, patient-centered approach [4]. The goal is to encourage more patients to be assessed and treated in primary care or within their local communities [4,5,6]. The current centralized testing systems are not a convenient process for many patients, owing to the disconnect between the testing and consultation process, frequently requiring more than a single visit to such a facility to complete the primary assessment/diagnostic process [6,7]. This testing process can be particularly unfavorable in LMICs, where availability, costs, travel times, and potential loss of daily earnings can be detrimental to patients, often resulting in follow-up visits not being fulfilled [7]. For such situations, rapid measurement strategies, in a home-/community clinic-based setting via PoC devices can play a key role in providing fast access to critical biomarker measurements and developing the care management pathway for the patients [2,6,7].

PoC devices have a long history, with the urine dipstick, developed in 1957, being one of the first of such devices [8]. However, it was not until the late 1960s when the development of lateral flow assay (LFA) technology and antibody-antigen immunoassay reactions gave the rapid diagnostic methods a real predictive value [2,9]. One of the main applications driving the development of rapid testing and PoC devices was the human pregnancy test, followed by the continual historical interest in urine testing for diagnostics [2,9]. In fact, it was the home-pregnancy test utilizing the lateral flow format which provided clear evidence of the value of the format for at-home use of antigen testing, while the rapid testing for the diagnosis of streptococcal pharyngitis (strep throat) popularized the LFA technology for the diagnosis of infectious diseases [2,10]. LFA technology has since come a long way with PoC devices now expanding into diverse disease areas including lifestyle (e.g., Type 2 diabetes), non-communicable as well as communicable infectious diseases (e.g., Influenza, HIV-1, HIV-2) [2,11].

The need for rapid, accurate diagnostics has been felt more than ever since the turn of this century. Since 2000 alone, there have been several major pandemics, including the 2003 Severe Acute Respiratory Syndrome (SARS) outbreak, the 2009 H1N1 influenza, the 2014 Ebola outbreak in Western/Sub-Saharan Africa, the 2017 Zika virus, and the most recent SARS-CoV-2 (COVID-19) outbreak, which has undoubtedly had the most impact globally [12]. Our ability to contain these diseases largely depends on our capabilities to not only identify their root cause but also to perform rapid diagnosis and deploy treatment to those who are affected [12]. As such, diagnostics is the first step in effectively identifying and managing the spread of epidemiological diseases. Unfortunately, as seen during the past years, the demand for effective diagnostics is often not met by an adequate supply and availability of resources. For example, The World Health Organisation (WHO) recommends nucleic acid amplification tests (NAAT) including the real-time reverse transcription (RT-PCR) for the identification of COVID-19 infection in triage and contact-tracing [13]. However, in resource-limited settings, such molecular testing is frequently only available in central reference laboratories [2,6,13]. Moreover, this testing capacity is limited, leading to long turnaround times which precludes its use for patient and infection control management in such scenarios [13]. To overcome this challenge, rapid diagnostic and PoC tests have been developed to help with the first-point mass-testing with advantages of short time-to-result, cost-saving, and alleviating pressure on central testing, albeit with varying specificity and sensitivity [13]. For instance, a high sensitivity (≥95% acceptable, ≥98% desirable) is required for COVID-19 contact tracing and diagnosis of cases with sub-acute infection, as both the viral load and pre-test probability (prevalence) are lower as compared to the triage setting of acute symptomatic patients [14]. However, for some of the popular COVID-19 PoC tests (CORIS COVID-19 Respi-Strip, BioEasy 2019-nCoV Antigen Rapid Test Kit) while their specificity was high (>99%), their sensitivity has been shown to range from 24–72% (low viral load) to 82–100% (high viral load), thereby limiting their use for patient care [13]. Nevertheless, these PoC tests have still been deployed in triage and contact tracing scenarios in Peru, Singapore, and United Kingdom, respectively. While this discussion around COVID-19 provides an example of the applicability of PoC tests and devices in large-scale monitoring of population during pandemics, there are a variety of other non-infectious and infectious diseases, which continue to pressurize the fragile healthcare systems in LMICs. While highly accurate diagnostic tests are available for infectious diseases of public health importance in the developed world, these tests are neither accessible nor affordable to the patients in resource-limited settings [2,6,7,11].

### 1.1. ASSURED, REASSURED Solutions and Current Bottlenecks

To alleviate the challenges faced in PoC testing in LMICs and to encourage the move towards benchmark standardization of PoC devices, the WHO published a set of guiding criteria in 2003 for an ideal test that can be used at all levels of the health care system, including resource-limited settings, to guide treatment and clinical management decisions [6,15]. These criteria are known by the acronym ASSURED-Affordable—Sensitive, Specific, User-friendly, Rapid and Robust, Equipment-free and Deliverable to end-users. While the ASSURED criteria are indeed difficult to meet in laboratory-based techniques of RT-PCR, enzyme-linked immunosorbent assay (ELISA), Western blot, and cell culture assays; paper-based lateral flow assays have the potential to meet many of these requirements [12,16]. Paper-based PoC devices [17,18,19], which expand on LFA technologies, offer a range of properties making them the favorable choice of technology platform for ASSURED devices [20]. These properties include [6]:

- Automated fluid handling, making devices cost-effective and easy to use;
- Ease of storage, transportation, and post-use disposal by incineration;
- The ability to perform multiplexed electrochemical and colorimetric assays;
- Visual colorimetric read-out using color change capabilities;
- Fabrication processes compatible with large-scale printing technologies.

Since 2003, several paper-based diagnostic tests have aligned well with the ASSURED criteria, including tests for HIV, malaria, syphilis, and tuberculosis [11]. Further use of electrochemical detection principles has led to the development of simple, portable techniques to analyze small molecules in a complex biological fluid environment with a high degree of specificity and sensitivity [21]. The integration of paper-based devices and printed electrodes has enabled low-cost diagnostics for the measurement of antibodies, metabolites, proteins, DNA, ascorbic acid, and metal ions for biological and environmental measurements [18,21,22,23]. Compared to colorimetric PoC devices, such paper-based electrochemical-microfluidic devices have been shown to offer higher sensitivity and selectivity, owing to enhanced mass transport on electrode surfaces which enhances the system current response [24,25]. However, such devices remain underexploited in field applications due to problems arising from ambient water absorption on paper and electrodes alike, compromising the system performance and is further limited by the requirements of skilled personnel and electrically powered expensive equipment (potentiostat/galvanostat) to run the electrochemical assay and enable quantitative read-out [21].

Similarly, the standard visual-/colorimetric-LFAs are subject to user bias arising from the time-bound colorimetric response and training [6,26]. In busy, trained-personnel limited or resource-limited clinical settings, these manual readouts hamper effective communication of the results, diagnosis, treatment, and prognosis. Additionally, the manual tests do not allow for effective communication of the result or diagnosis, or data collection for assessment of epidemics or therapy strategies [6,11,26]. A more sophisticated quantitative read-out, as compared to a binary yes/no response, necessitates some form of external instrumentation to perform processing and communication. In the case of resource-limited/remote settings, the option of using proprietary LFA strips, cassettes, and a PoC reader (the most expensive component of the setup) is unfeasible due to cost and accessibility constraints. The limited network and maintenance infrastructure, intermittent power, and issues with reliable, controlled storage further create a unique set of challenges for the successful implementation of any such instrumented solution. Capturing, conveying, and storing the test results via automated result readout and communication would contribute to traceability in the healthcare system. To facilitate the effective implementation of read-out, displays, and communication modules, on-board power is therefore a necessity to drive these added functionalities, without relying on external power sources. Thus, there is a clear and present need for quantitative detection, read-out, and connectivity to be integrated into an automated, self-powered, maintenance-free system, ideally incorporated on the PoC device itself [26]. Considering these challenges and unmet needs, a fully ASSURED and next-generation REASSURED device, which incorporates Real-time connectivity, Ease of sample collection, and Environmentally friendly aspects [11], would therefore require further integration of the following functional components [6]:

- Actuation and control modules for operation of and user interaction with the device, improving usability and providing built-in instrumentation;
- Microfluidics for processing and fluidic control of the sample to be tested, contributing to low-cost diagnostics with high specificity;
- Electronics to add sensitivity and speed towards automated, integrated testing and instrumentation;
- Sensing modules towards built-in, fast, and sensitive detection techniques and instrumentation;
- Data processing capabilities to analyze the sample accurately and automatically and capture the result digitally, contributing to most aspects of ASSURED;
- Readout and display modules to express the result directly to the user, improving result read-out times and user-friendliness with built-in instrumentation;
- Connectivity for transmission and storage of results to be accessed remotely or in future as needed, contributing to many ASSURED aspects, and
- Energy storage/generation for built-in power to drive the various functional components, assisting with deployment of devices with on-board instrumentation.

The above functional components also align well with the recently developed STARLITE (Sample-to-answer, Rapid, Local, Inexpensive, Throughput/Equipment) criteria, specifically formulated for the design of PoC tests for pandemics [27]. In all cases, the integration of power to drive PoC solutions is crucial, as several other functional components rely on power to operate. Self-powered devices are of particular importance, as in resource-limited settings, power supplies cannot be relied on [26]. In the long-term, traditional batteries provide a compact power solution but introduce challenges in terms of environmental friendliness, which is an important REASSURED consideration. Energy harvesting is a powerful tool that can be harnessed to drive PoC diagnostics, particularly in under-resourced settings, and has been demonstrated to some extent in the literature [21,28,29,30]. Some of the key aspects of the energy requirements for diagnostic devices are discussed in the next section.

### 1.2. Energy Requirements and Self-Powering of Diagnostic Devices

In 2016, Choi [31] had reviewed the powering mechanism of PoC diagnostic devices considering power-free, battery-powered, and smartphone-based devices and projected a convergence of disposable paper-based devices, reusable handheld systems, and mobile healthcare technologies. Concurrently, as discussed in the previous section, the move towards patient-centric care, continuous high-accuracy measurements, and the need for quantitative read-outs necessitates the development of self-powered, autonomous monitoring. For both biomarker and physiological signals and their continuous or intermittent monitoring, the following major challenges currently exist: (i) improvement of physiological signal measurement accuracy, which is currently affected by noise artifacts, (ii) improvement of selectivity and sensitivity especially for multiplexed measurements, and finally (iii) overcoming the high-power consumption of such devices especially during continuous monitoring [32]. For quantitative biomarker measurements, the biomolecular interactions resulting in a signal are further transduced via either optical (colorimetric, fluorescent, luminescent, interferometric, etc.) or electrochemical methods (amperometry, voltammetry), resulting in higher sensitivity and resolution [31]. These require mW-powered light sources such as light-emitting diodes (LEDs) or low-cost/-power (mW) portable galvanostats compatible with portable readers [31]. Similarly, the improvement in physiological signal analysis requires either improvement in the signal capture mechanism or digital signal processing tools, which again necessitate on-board power.

**Figure 1:**
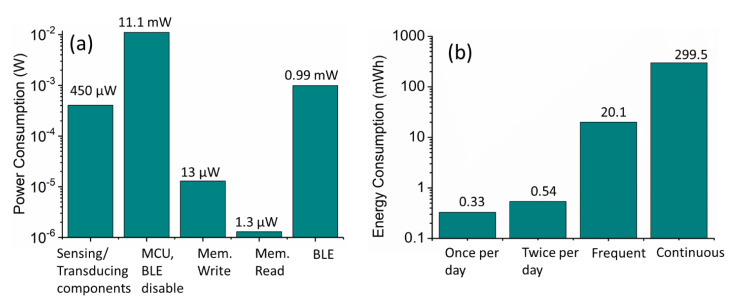
(**a**) the energy consumption of the key components in a wireless sensor system monitoring PPG, ECG, and temperature simultaneously, (**b**) Energy consumption in a day for “continuous measurement,” “frequent measurement,” and “measurement once or twice a day.” Figure adapted from Yu et al. [32].

A typical device for such biomarker/physiological measurements is usually implemented with the following blocks: sensing, signal acquisition/processing, microcontroller with a memory module, read-out display, and/or communication modules (Table 1). Out of these, the sensing front-end is the least power-hungry (nW-10 s of μW of power) [32,33]. Although optical detection-based devices such as photoplethysmography (PPG) and PoC readers consume significantly higher power (10 s–100 s of mW, depending upon the current), their consumption can be significantly reduced to circa 100 μW level by controlling LED operation via a short pulse (10 s of μs) duration duty cycle [31]. The complexity of the next stage of the pre-processing circuit, i.e., signal acquisition incorporating interface, signal-conditioning, amplification, filtering, and analog to digital (A/D) conversion determines its power consumption, and application-specific integrated circuits (ASICs) can be optimized to consume as low as tens to hundreds of μW of power. For example, an ASIC integrating low-power analog front end and a digital back end for PPG monitoring was shown to consume ~172 μW of energy [34]. As compared to the sensing and pre-processing circuits, the data processing (microcontroller units (MCUs)), storage (memory), and transmission (Wi-Fi, Bluetooth) block of the system architecture is the most power-hungry element and provides the largest obstacle towards self-powered, end-to-end solutions. For example, while the continuous operation of MCU requires mW level power, the “read”, “write” and “reset” memory operations are typically less energy-intensive and require μW level power [35]. Comparatively, the wireless communication protocols and devices are extremely energy-intensive requiring 10 s of mWh for sending and receiving data, which can and have been reduced via a shortening of the duty cycle and data packet-size [35]. For example, a typical Bluetooth Low Energy (BLE) module which is mostly in deep sleep mode during the cycle, when activated to transmit 900 bytes of data in a 60 ms duty cycle will consume around 0.99 mW of power [32]. When considered in conjunction with a typical PPG, electrocardiogram (ECG), or a temperature sensor, a 1-s cycle will typically consume ~12.5 mW of power (see Figure 1a,b).

To analyze and achieve an entirely self-powered system, the continuous measurement and evaluation requirements of the physiological or biomarker parameters need to be established. In the case of a high-risk disease such as cardiovascular condition, there is a need to continuously monitor the subject’s parameters including ECG, heart rate, variability and even breathing patterns (PPG). Whereas for certain sleeping disorders, there is a case for frequent (say every 10–15 min during sleeping) but non-continuous monitoring of the respiratory parameters. In the case of blood sugar measurements and monitoring, the measurement frequency is lower at perhaps 3–4 times a day. Considering each of these scenarios, the consequent energy/power demands of the system would be significantly different from each other (Figure 1b). For example: in the case of continuous heart rate/respiratory monitoring, the typical energy consumption is ~12.5 mWh [32]. Yu et al. [32], have estimated that even for the best performing flexible Li^+^ batteries, the system can only operate for ~65 h before charging is required. Even when the system is supported by an energy harvesting module, which can trickle-charge the battery, the system’s operation time can only be extended by another 6 h to 71 h. For the frequent but non-continuous measurements, while the power consumption will remain similar the turn-on time is significantly reduced, thus providing a much better balance between the energy demand and supply. For example, at an hourly energy consumption of 0.84 mWh (corresponding to a reading every 15 min), the harvested energy of 26.5 mWh is sufficient to extend the system operation time to around 31.5 h. Therefore, if the battery capacity is higher than the daily harvested energy and can sustain the device’s operation for the first day, the subsequent energy harvesting will ensure that the system can become self-powered. Finally, considering the “once/twice a day” infrequent measurements the energy consumption is ~0.5 mWh which may potentially be powered entirely by the energy harvesting device itself.

Considering these power budgets, a variety of experimental devices based on various energy harvesting mechanisms are available including thermoelectric, photovoltaic, piezoelectric, and more recently triboelectric effects [28,29,36]. While extensive reviews on triboelectric sensors, systems, and materials are available [37,38,39], from the reader’s perspective, it would be beneficial to provide essential background information on triboelectric effect and triboelectric nanogenerator (TENG) devices. The triboelectric effect is a combination of contact electrification and electrostatic induction phenomenon. The contact charging/contact electrification occurs when two materials of typically significant differences in their charge donation/acquisition properties, are brought into contact with or rubbed against each other. This leads to a charge exchange between the two materials therefore causing the two surfaces to acquire equivalent but opposite charges [38,39,40,41]. These immobile surface charges then induce electronic charge in the metal back-electrodes via electrostatic induction. Since the first report of the TENGs in 2012 [40] these devices have gained worldwide attention in the fields of both energy harvesting and self-powered sensing [37,38,39]. The theory and working principle for the different types of the standard TENG devices, classified by their operating modes, are explained briefly in the next section.

#### 1.2.1. Vertical Contact-Separation-Mode TENGs

**Figure 2:**
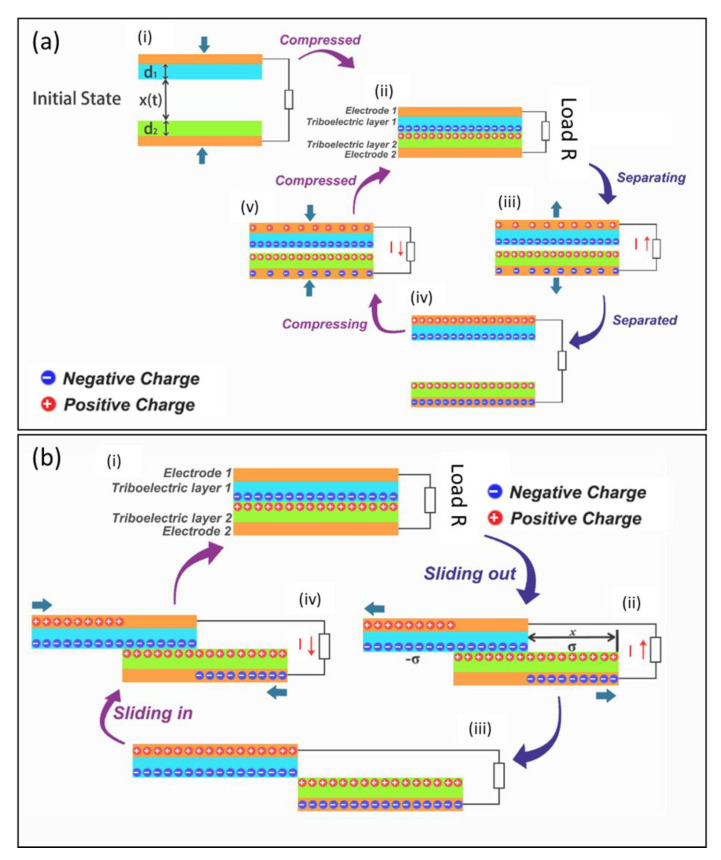
(**a**) Schematic of one complete charge generation cycle of a vertical contact-mode Triboelectric Nanogenerator (TENG), and (**b**) schematic for a standard parallel sliding-mode triboelectric nanogenerator.

The standard vertical contact-separation-mode TENG operates via the periodical vertical contact-separation mechanical motions induced varying electric field between two tribo-layers [40,41]. The induced potential built on backside metallic electrodes can be used to drive alternating charge flow in the external circuit connected between the two electrodes. A brief demonstration of such a TENG’s energy harvesting cycle is shown in Figure 2a. In the initial state (Figure 2(ai)), there is no charge or induced electric potential difference between the two electrodes. When two triboelectric layers are bought into contact by an external force (Figure 2(aii)), the surface charge transfer takes place due to the triboelectrification effect. An electric field is generated between two contact layers during the subsequent release process which induces an electric potential between the back-electrodes which drive electrons to flow from the bottom electrode to the top one (Figure 2(aiii)).The strength of the electric field keeps on increasing until the layers are fully released to their original position (spacer distance) and the electric field reaches its maximum values (Figure 2(aiv)). At this position, the system reaches an electrostatic equilibrium state. Subsequently, when a pressing force is then applied to make two layers to come close again, the reverse change of the electric field strength will induce a potential with opposite direction to drive the electrons flow back from top electrode back to bottom one (Figure 2(aiv)) until two layers fully contact with each other to reach another equilibrium state (Figure 2(aii)).Thus, when two electrodes are connected, each generation cycle of a typical vertical contact-mode TENG device will generate a complete alternating electrical signal.

Owing to the system design requirements, it is necessary to have a complete theoretical understanding of the energy harvesting process. Niu et al. developed a comprehensive theoretical model to understand and clarify the relationship of the real-time output performance and the main parameters of the TENG devices [41]. As shown in Figure 2a, the TENG structure can be viewed as a parallel plate capacitor system with two back-electrodes (electrode 1 and electrode 2), and dielectric contact layers (triboelectric layers) of the thicknesses *d*_1_ and *d*_2_, respectively. In the vertical contact-mode TENG system, the most critical output and dynamically controlled operation parameters are:

- The voltage (*V*) generated between two electrodes by charge induction;
- The amount of transferred charge (*Q*) between two electrodes;
- The separation distance (*x*) between two triboelectric layers (*x(t)*) representing the separation distance during real operation time *t*.

The system with a tribo-contact-area of *S* can be modelled by defining the relationship between these three parameters, which was named as *V-Q-x* relationship [41]. Under ideal conditions, without considering the non-uniform distribution of the electric field on the edges, the open-circuit output voltage (*V_oc_*) established between the back electrodes can be expressed by:

(1) $$V_{oc}=\frac{\sigma x\left(t\right)}{\varepsilon_{0}}$$

where $\varepsilon_{0}$ represents the difference in permittivity between a vacuum and air.

At the short-circuit conditions, a charge flow is driven through the connecting wire between the two electrodes via the induced potential. The amount of transferred charge $Q_{sc}$ can be presented by:

(2) $$Q_{sc}=\frac{S\sigma x\left(t\right)}{d_{0}+x\left(t\right)}$$

Thus, the generated *I_SC_* is given by:

(3) $$I_{sc}=\frac{dQ_{SC}}{dt}=\frac{S\sigma d_{0}v\left(t\right)}{{\left(d_{0}+x\left(t\right)\right)}^{2}}$$

where

(4) $$d_{0}=\frac{d_{1}}{\varepsilon_{1}}+\frac{d_{2}}{\varepsilon_{2}}$$

Here, *ε*_1_ and *ε*_2_ are the relative dielectric constants of two triboelectric contact layers. By observing these established models, the generated potential difference, transferred charge, and resulting current are all directly proportional to the surface charge density (*σ*) generated during the contact charging process. Thus, it is reasonable to suggest that the improvement of the surface charge density, *σ*, on the contact interface is the key to enhancing the output performance of TENGs [37,38,39,42].

#### 1.2.2. Sliding-Mode Triboelectric Nanogenerators

The parallel sliding-mode TENGs operate via an in-plane sliding motion occurring between the two in-contact tribo-layers. As shown in the schematic of the energy generation cycle (Figure 2b), the periodical change of the contact area between two sliding layers leads to an alternating flow of induced charges on the electrodes to drive electrons across the connected external load [43,44]. When the two triboelectric layers are fully overlapped and in contact with each other, like the contact-mode TENG, the surface charge transfer takes place due to the triboelectrification effect. Net positive charges will be generated on the surface of the tribo-positive layer, and the same amount of negative charges will be transferred to the surface of the tribo-negative one (Figure 2(bi)). When the two layers start to slide parallelly to separate, an increasing electric field with the direction pointing from left to right almost parallel to the sliding plane is generated to induce a growing potential on the back electrodes (Figure 2(bii)). In the meantime, a current flow is driven from the bottom electrode to the top one to balance the potential difference until two plates are completely apart or the sliding motion is stopped (Figure 2(biii)). A reverse sliding motion will cause a reversal of the generated electric field and induce a potential between back electrodes in the opposite direction (Figure 2(biv)). Thus, the electrons will now be driven back from the top electrode to the bottom one to maintain the electrostatic equilibrium until two layers are slide back to the original position (Figure 2(bi)) [43,44].

To simplify the analysis and design of the sliding-mode TENGs, a theoretical model has been developed [44], wherein, the *V-Q-x* (where *x* represents the relative parallel separation distance in the case of a sliding-mode TENG) relationship and the resulted short-circuit current (*I_sc_*) can be expressed by:

(5) $$V_{oc}=\frac{\sigma x\left(t\right)d_{0}}{\varepsilon_{0}\left(l-x\left(t\right)\right)}$$

(6) $$Q_{sc}=\sigma x\left(t\right)$$

(7) $$I_{sc}=\sigma w\frac{dx}{dt}$$

where *l* is the length of the sliding layers in the direction of sliding, *w* represents the width of the sliding layers in the direction perpendicular to the sliding direction, *ε*_0_ represents the difference in permittivity between vacuum and air, and the value of *d*_0_ is a constant parameter of the produced device which can be calculated by Equation (4). As is the case with the vertical contact-mode TENGs, all the output performance parameters are directly proportional to the surface generated tribo-charge density (*σ*).

Given the advantages offered by triboelectric nanogenerators (TENGs), including simple structure comprising of readily available materials [45,46,47], flexibility [45,46], low-cost [48], lightweight [49,50], high efficiency [50,51], biocompatible/environmentally friendly and most importantly, the high-power density [45,50,52], this review focusses on TENG-based devices only. All these aspects make TENGs particularly suitable for developing REASSURED solutions. The potential of TENGs for wearable and implantable health solutions has recently been showcased [53,54,55] and provides a foundation on which to demonstrate the use of these devices for developing REASSURED PoC solutions. This review aims to provide the reader with the current state of the art around self-powered healthcare and PoC devices. In view of the increasing number of wearable healthcare devices, we believe this review will provide a comprehensive overview of the current state of the technology and will prove useful in designing new strategies by building upon the current knowledge base. We further discuss the potential applications, challenges, and our perspective on self-powered PoC devices.

## 2. Current State of the Art of Self-Powered PoC Sensors and Devices

Healthcare diagnostics and management has been helped immensely by the breakthroughs in biosensors which rely on biochemical interactions between a sensor and an analyte with high specificity [56]. Comprehensive reviews on flexible and wearable biosensors for health [56,57,58] highlight the move towards solutions that are well aligned with the REASSURED principles. These sensors require power to function effectively as integrated solutions. The following sections provide an overview of self-powered sensing solutions, utilizing TENG devices, which can be leveraged to realize REASSURED PoC health diagnostics and monitoring solutions.

Self-powered TENG electrochemical sensors, along with self-powered flexible and paper-based TENG devices for electrochemical and colorimetric readout towards the long-term REASSURED goal are discussed. Following this, self-powered monitoring solutions using TENG devices for cardiovascular, respiratory, and other important physiological signals are presented.

### 2.1. Self-Powered Electrochemical Sensors

In one of the earliest reports, Zhang et al. [59] presented a contact-separation TENG based on a patterned polydimethylsiloxane (PDMS) film and Aluminum electrode for harvesting biomechanical energy from human walking. Under general walking conditions, the 2 × 7 × 0.08 cm single-layer TENG stuck on the subject’s clothes produced ~25 *V_p-p_* (peak-to-peak) and a current density of 0.02 μA·cm^−2^. As the alternating current (AC) pulse generated by the TENG is unsuitable for directly driving the CuO-based glucose sensor, the TENG’s output was utilized to trickle-charge a Li^+^ battery which then drove the sensor. While a good linearity range (0.1–1 mM) was observed for the sensor, the device required more than 2 h of clapping at a 2 Hz frequency to charge up the battery from 440 to 800 mV (the potential required for electrochemical sensing of glucose) [59]. Chen et al. [55] proposed a self-powered lactate sensor based on a gelatin-PDMS-based TENG. The sensing layer of electrochemically deposited Pd nanoparticles on carbon fibers was deposited using the TENG itself on which a thin chitosan layer was coated to protect the electrode. The TENG was able to provide a maximum output voltage of 500 V and a current density of 14 mA·m^−2^. This electrical output was fed into a full-wave rectifier to charge up a 1000 μF capacitor to 5 V in only 30 min under an operational frequency of 1 Hz. The self-powered lactate sensor’s current response increased linearly (10 µM to 20 mM range), while in the case of sweat analysis (diluted with a phosphate-buffered saline (PBS) solution) a value of 11.75 mM was calculated which fell within the healthy human range. The power generated from the TENG could charge the capacitor sufficiently to enable lactate measurement every 30 s. 

Extending the work further, He et al., developed a self-powered E-skin for real-time perspiration analysis using Polyaniline (PANI) triboelectric-biosensing unit matrix [60]. The E-skin’s working mechanism is based on the solid-water surface-based triboelectrification/enzymatic-reaction coupling between PANI and water. The 2.8 × 3.4 cm^2^ E-skin device with each biosensing unit of 0.5 × 0.5 cm^2^ area was shown to detect urea, uric acid, lactate, glucose, and Na^+^, K^+^ concentration in perspiration. A patterned Cu network (3 cm × 4 cm × 10 μm) working as an electrode was located in a groove-patterned flexible PDMS layer (prepared via photolithography and wet-etching) and worked as an electrode while PANI deposited on the Cu network by electrochemical polymerization acts as the frictional material. On the back surface of the PDMS layer, an evaporated Cu film (thickness ~100 nm) works as the other electrode. The triboelectrification effect between PANI and water can be influenced by the products of the surface enzymatic reaction through changing the surface chemical state of PANI which then leads to a change in the current output of the TENG from each of the sensing units. While a variation in the output characteristics (reduction in current) with different analytes was observed, a calibration curve could be utilized to directly analyze the perspiration by attaching the E-skin device to the elbow, without any external power. This E-skin represents a highly promising platform for sweat analysis, although presently the measurement of all analytes varied considerably against a control measurement after 30 min of exercise. Detailed studies on sweat sampling by Peng et al. [61] and Twine et al. [62] of the same group, which try to address the difficulties encountered during the accurate electrochemical measurements of sweat, such as contamination by hair and skin, suggest that future TENG-powered E-skin devices can obtain a higher level of accuracy.

Zhao et al. presented a self-powered biosensing E-skin for real-time Ca^2+^ detection in sweat along with wireless data transmission capabilities [63]. Based on the triboelectrification/enzymatic-reaction coupling effect (PDMS-water system), the self-powered E-skin was shown to harvest tiny amounts of mechanical energy from human motion and directly output biosensing signals. The active layer of PANI (with nicotinamide adenine dinucleotide phosphate oxidase 5 (NOX5) and nicotinamide adenine dinucleotide phosphate (NADPH) modifications) displayed a much steeper increasing Ca^2+^-dependent output triboelectric current (2.2 × 10^−1^ G·L^−1^ Ca^2+^) which was much higher than the control PANI (without modification). The 2 × 5 cm device outputted a voltage of 3.9 V with a current of 1.7 μA and was shown to power up a wireless transmitter to send the biosensing information. Though, it should be noted that the wireless receiver was powered up using conventional batteries only.

Jiang et al. demonstrated how a TENG device that utilizes a liquid-solid contact in an oil/water multiphase system can be used to provide a self-powered biochemical sensing platform for dopamine (DA), an important neurotransmitter which is a biomarker in conditions such as Parkinson’s disease and schizophrenia [64]. The single-electrode TENG was fabricated with a polytetrafluoroethylene (PTFE) film, copper electrode, and a glass substrate. The TENG device generates two current signals upon insertion into an oil/water multiphase liquid: the first signal is a current caused by the electrostatic induction between the PTFE film and liquid, and a second interface current caused by interfacial charges at the interface of the oil/water. To use the TENG as a sensor for DA, the device is immersed in a liquid containing DA, whereby it polymerizes to form a polydopamine (PDA) onto the PTFE and glass surfaces of the device. The PDA on the TENG surfaces alters both the electrostatic and interface currents measured as the TENG is immersed in the oil/water multiphase. As the measured current for both signals depends on the concentration of PDA on the TENG surfaces, the current can be used to determine the concentration of the analyte. The ability to measure the analyte concentration using two separate current readings could help to minimize any false readings that can occur in a complex biological liquid. Whilst this novel technique shows great promise as a self-powered sensing platform, some hurdles remain to be overcome. The TENG device was immersed in the DA solution followed by stirring for 12 h, which may limit the throughput of the device compared to other types of electrochemical measurement and could be affected by biofouling during this time in a complex matrix. This sensor principle also appears to be limited to those analytes which readily polymerize on the TENG surface, currently limiting its applicability.

The examples listed above utilize human movement in the form of walking or running to harvest energy, where the devices are typically stuck to human clothing (Zhang et al. [59], Chen et al. [55]) or onto the skin directly (He et al. [60], Zhao et al. [63]). Glucose sensing and lactate sensing were both successfully shown by Zhang et al. [59] and Chen et al. [56], respectively. However, the PDMS-based TENG devices alone were not sufficient to power the sensors and were required to charge external powering devices. The work of Zhang et al. [59] required the TENG to run for more than 2 h to sufficiently charge a Li^+^ battery for adequate sensing. For lactate sensing showcased by Chen et al. [55], the TENG took 30 min to effectively charge a capacitor to enable lactate measurements to be taken every 30 s.

Advances where external power sources were not required were also explored using triboelectrification/enzymatic reaction coupling for sweat analysis (He et al. [60], Zhao et al. [63]), with the latter enabling wireless transmission capabilities and improved sensing accuracies. Sensing of dopamine—having far reaching applications—was exhibited by Jiang et al. [64] but this approach required the TENG device to be immersed in a DA solution with mixing for 12 h, limiting the throughput and analytes that can be detected.

**Fulfilling REASSURED criteria:** The skin-mounted self-powered sensors such as the E-skin TENGs provide “Ease of sample collection”, eliminating the need for specialized sample collection procedures.

#### Flexible and Paper-Based Sensor Devices

**Figure 3:**
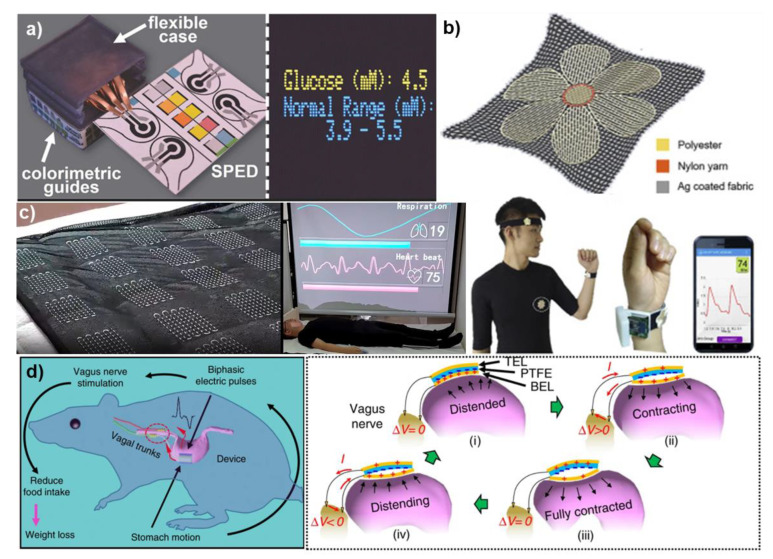
Examples of TENG-powered sensor applications. (**a**) A self-powered electrochemical device based on a paper microfluidic sensor is integrated with a potentiostat to give a user-friendly glucose reading, figure adapted from [21], (**b**) A flexible, textile-based pressure sensor is used as a user-friendly wearable device for monitoring pulse wave signals and wirelessly transmitting them figure adapted from [65]. (**c**) A smart textile sensing unit uses triboelectrification and electrostatic induction to monitor respiration and heart rate during sleep, figure adapted from [64]. (**d**) An implanted vagus nerve stimulation system is biocompatible and self-powered. As the stomach is enlarged, this triggers a current along the vagus nerve which helps to control the overeating, figure adapted from [63].

**Figure 4:**
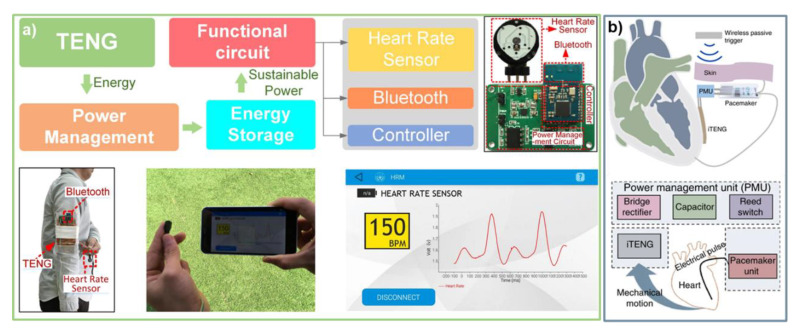
Examples of complete TENG powered systems. (**a**) A downy-structured TENG is used in a system where a capacitor is charged by a wearable device, and used to power a sensor, controller, and communication system [72]. (**b**) An implanted triboelectric pacemaker, including a controller and power management system, is powered by the heart motion [79]. Figures adapted from Lin et al. [72] and Ouyang et al. [80], respectively.

In their work, Pal et al. have reported on the fabrication of self-powered, paper-based electrochemical devices (SPEDs) using cellulose paper with patterned hydrophobic domains that delineate hydrophilic, wicking-based microfluidic channels for accurate colorimetric assays, and self-pipetting test zones for electrochemical detection [21]. The use of hydrophobic paper for the construction makes the electronic components and contact electrodes stable over a wide range of relative humidity and even after wetting the device with biological samples (see Figure 4). The power for these SPEDs is generated via a TENG employing a paper-PTFE triboelectric pair with Ni back-electrodes (see Figure 3). Under mechanical excitation, the TENG provides a peak-to-peak voltage of up to 400 *V_p-p_* and a current of 59 μA, respectively. At the impedance matching load of 1.5 MΩ, a maximum power of ~63 μW·cm^−2^ could be delivered which was used to store charge (after rectification) in a 47 μF capacitor to trickle charge a Lithium battery which then enabled three-electrode electrochemical measurements based on an LMP91000 low-power sensor. The screen-printed carbon and Ag/AgCl electrodes showed fast electron transfer kinetics for Fe (CN)_6_^3−/4−^ redox system with excellent linearity (R^2^ = 0.99) of anodic/cathodic peak currents against the square root of the scan rate (2.5 to 15 Mv·s^−1^, −0.3 to 0.7 V potential window) and reproducibility (standard deviation of 3.6% across 10 different SPEDs). When utilized for the measurement of buffered solutions of potassium ferrocyanide (redox model compound), glucose, uric acid, and l-lactate, the SPED based quantification via chrono-coulometry was more accurate than chronoamperometry, especially for small (<0.2 × 10^−3^ M) concentrations of analytes (faradaic currents <300 nA, test time ~6 s), due to the experimental noise peaks registered by the portable potentiostat. As such, the limit of detection (LoD) for glucose, uric acid, and l-lactate using SPEDs and portable potentiostat in the chrono-coulometry configuration is 0.2 × 10^−3^ M, which is within the typical cut-off value range of all these analytes. 

Besides running these accurate, quantitative electrochemical tests, the authors utilized SPEDs for colorimetric analysis for leukocyte, nitrite, urobilinogen, protein, pH, hemoglobin, specific gravity, ketone, bilirubin, and glucose in urine [21]. These ten colorimetric test zones included in the middle of the SPED to increase the surface area of the device and the consequential triboelectric charge density of the TENG, demonstrated the compatibility of SPEDs with conventional, wicking-based, paper microfluidics applications which require the sequential wetting of test zones. A low-power-consumption, fast (<25 s) machine-vision algorithm was also developed to automatically identify and quantify each of the colorimetric test zones from a digital image. To provide fast diagnostic results to the user and to potentially facilitate remote expert consultation, the algorithm’s accuracy was tested for both low (240 × 320 pixels) and high resolution (2448 × 3264 pixels) images as well as varying environmental illumination (40–75,000 Lux) and orientations (0°–355° in-plane; 0°–30° out of plane) and shown to work efficiently by obtaining a standard deviation of the recognized colors of 0.92% and 2.96%, respectively. Thus, the integrated paper-based SPEDs can not only provide sensitive, accurate, quantitative measurements but can also power up other devices to facilitate telemedicine applications. 

While there is no doubt that the PoCs based on self-powered, paper-based electrochemical devices offer significant advantages over other devices, the SPEDs face limitations too, including:

- The TENGs currently interfaced to the SPEDs require the user to tap the device with three fingers at 2 Hz for ~4 min to provide the portable potentiostat with enough energy to perform a 28 s electrochemical test;
- To complete multiple (currently up to four) electrochemical measurements, the position of the electrodes needs to be manually shifted to a particular SPED, i.e., only one electrochemical assay can be performed at a given time.

**Fulfilling REASSURED criteria:** Paper-based solutions are poised to assist with “E”—environmentally friendly aspects while the self-pipetting properties offer “Ease of sample collection”. Rapid solutions like SPEDs are “Deliverable to end-users” at a low-cost while offering multiplexing capabilities.

### 2.2. Self-Powered Monitoring of Electrophysiological Signals

Electrophysiological parameters, such as respiratory rate, heart rhythm, beats per minute (bpm), and blood pressure, represent some of the most valuable and vital clinical diagnostics [57,65]. The absence of timely detection of sudden and transient changes in these can often result in the development of life-threatening conditions. While for in-patients, intermittent measurements are carried out; for at-home patients, the use of bulky recorders offers a significant challenge due to the discomfort, complexity, and even inadequate patient compliance [66]. The emergence of wearable-devices with some of them being implantable devices provides a pathway for continuous, stable, and real-time measurements required for reliable diagnosis and intervention for patients’ severe diseases [54,57]. The development of self-powered, non-invasive solutions which can be integrated into an individual’s life via means of wearables—textile-based or even dermal—can provide pathways to monitor vital signs in real-time which are discussed below. Within the next sections, the emergence of self-powered measurements of electrophysiological signals and other vital signs will be discussed.

#### 2.2.1. Cardiovascular Monitoring

Cardiology represents one of the most popular applications for self-powered sensors and devices, with a significant number of publications and reviews in the area [65,66,67,68]. The arterial pulse wave which is often the most accessible and representative biomedical signal, carries a significant amount of information about the cardiovascular system’s condition [69,70]. The extraction of pathological and physiological information from the arterial pulse wave for cardiovascular diagnosis is clinically carried out using PPG and piezoelectric pulse transducer (PPT) techniques [71]. While the PPG is highly sensitive to body movement and ambient light variation, the PPT is limited owing to its high cost and relatively lower sensitivity. As is the case for all the other sensors, the energy consumption and battery requirements have provided a constant challenge for miniaturization and weight reduction of these commercial techniques [65,67,71]. Thus, in the future, a low-cost, non-invasive, self-powered, and user-friendly heart monitoring system is highly desirable, and triboelectric sensors and devices can enable these features [72]. In the literature [65], the research into self-powered cardiovascular devices can be broadly divided into two categories: (i) the use of TENGs to harvest biomechanical/ambient energy for powering monitoring devices, and (ii) the use of TENGs as active sensors for monitoring.

Wang’s group’s pioneering work [73] in energy-harvesting TENGs and their applications led to the development of multi-layered vertical contact-separation mode TENG comprising of a Kapton (polyimide, PI) film backbone architecture with Al and nanostructured fluorinated ethylene propylene (FEP) with a 300 nm Cr/Cu back electrode, as the triboelectric pair. Through the simultaneous development of efficient (~60%) power management circuits and low-leakage storage, such universal self-charging systems driven by low-frequency (2 Hz) body motion are capable of sustaining operation of mobile and wearable electronics, such as temperature sensors, heart rate monitoring devices, pedometers, wearable watches, and radio-frequency wireless transmitters. When integrated into a shoe insole, the Al/FEP TENG produced an output voltage of 700 V, while when excited with palm tapping at 1.6 Hz frequency, the TENG was shown to be capable of maintaining continuous operation of a heart rate and temperature sensor.

One of the earliest examples of the use of TENGs in the measurement of cardiovascular parameters was reported in 2014 by Zheng et al. [74]. The fabrication of their implantable TENG (iTENG) was carried out using PDMS films with patterned pyramid arrays (50 nm gold back-electrode) and nanostructured aluminum foil with a 400 μm flexible polyethylene terephthalate (PET) spacer. The device was further encapsulated in a 50 µm PDMS layer to isolate it from the surrounding physiological medium and to ensure biocompatibility when implanted into an adult rat. The iTENG harvested energy from periodic breathing (mechanical deformation related to the periodic expansion and contraction of the thorax) to generate an AC output voltage of 3.7 V and a short-circuit current of 0.14 μA. More importantly, the converted electricity could be stored in a capacitor and used to power a 555 timer-circuit to output a continuous stream of rectangular pulses at a specified frequency. The iTENG was shown to be able to charge the pacemaker capacitor to 2–3 V within 275 min (a total of 13,750 breathing cycles). In 2016, the same group proposed improvements and implanted the device into large animals for the first time [75]. Driven by the heartbeat of an adult swine, the output voltage, and current reached values of 14 V and 5 μA, which were nearly 3.5 and 25 times higher than the initial reports [74]. The in vivo demonstration of the iTENG device for over 72 h after implantation proved the continuous production of electricity in an active animal. This long-term reliability was further complemented by the development of a self-powered wireless transmission system wherein the electrical signal associated with the in vivo heartbeat was successfully transmitted, showing its feasibility for real-time remote cardiac monitoring.

Ma et al., have reported on self-powered cardiac monitoring, and continuous monitoring of multiple physiological signs [66]. The flexible implantable triboelectric active sensor (iTEAS) comprised of a Kapton film as the substrate on which nanostructured-PTFE (n-PTFE) film was fixed, which then contacted an aluminum film (acting as the second electrode). To ensure the vertical separation between the two surfaces, spacers comprising of an elastic titanium strip were integrated on Kapton film. As the device was to be used in vivo, medical-grade packaging comprising of PTFE, PDMS, and Parylene films was carried out to avoid potential erosion in the physiological environment. The flexible iTEAS was implanted into the pericardial sac of a living swine and fixed to the pericardium. In response to the heartbeat and breathing, the iTEAS produced a voltage of ~10 V and a short-circuit current of ~4 μA and enabled the detections of tiny changes of motions of circumferential organs. A comparative analysis of the standard ECG and iTEAS device showed excellent compatibility (~99% accuracy in measuring the heart rate), however, the synchronicity was only measured for 72 h. Similarly, the respiratory rate could be ascertained from the iTEAS by monitoring the motion and voltage output at various locations including the left lateral wall (LLW), right lateral wall (RLW), and posterior wall (PW) of the heart. At an artificially induced ventilating rate of 12 cycles per minute, the LLW provided the most stable and accurate output of time interval between the two neighboring maximal peaks of ~ 5 s. However, as compared to the body-worn sensors, there is no doubt that the challenges for such in vivo devices are much larger. For example: (i) hermetic bio-compatible packaging needs to be guaranteed to ensure the performance of the device and no leakage of physiological fluids into the device, (ii) the thickness of the encapsulation layer needs to be strictly controlled to maintain the sensitivity of medical implants in responding to biomechanical motions and finally (iii) flexibility of the iTEAS devices is required to ensure conformability to organs inside the body.

Liu et al. [76] have reported on the development of a self-powered triboelectric endocardial pressure sensor for monitoring of heart failure patients with impaired cardiac function. The long-term, continuous data collection of endocardial pressure is usually captured through invasive and expensive cardiac catheterization. The device of 1 cm × 1.5 cm × 0.1 cm employed corona-charged nanostructured PTFE film against Al foil as the triboelectric pair with an ethylene-vinyl acetate (EVA) copolymer film as the spacer layer and was further encapsulated by PDMS to ensure leakproof behavior. The real-time biomedical evaluation of the device was carried out in a male adult Yorkshire pig where it was implanted in the left ventricle and the left atrium. Working signals of ECG, femoral arterial pressure (FAP), and endocardial pressure, were measured and compared under resting, arousing, and active conditions. At the resting status (systolic FAP < 100 mmHg), a positive *V*_oc_ of ≈ 80 mV was obtained, with both FAP and *V*_oc_ remaining constant, indicating that the TENG device exerted little loading on the cardiac functionality. The TENG device was shown to achieve high-sensitivity (1.195 mV·mmHg^−1^), real-time monitoring, and long-term stability (more than 100 million working cycles) and shown to detect cardiac conditions such as ventricular fibrillation and ventricular premature contraction.

Utilizing biodegradable polymers of poly (L-lactide-co-glycolide acid) (PLGA), poly (3-hydroxybutyric acid-co-3-hydroxyvaleric acid) (PHB/V), poly (caprolactone) (PCL), and poly (vinyl alcohol) (PVA), and conducting layer of magnesium, Zheng et al. [77] reported development of a unique, fully resorbable transient electronics-based TENG. Both in vitro and in vivo (in rat) biodegradation studies were performed to investigate device performance, absorption, and transient behavior. The in vitro studies of PLGA coated devices showed that significant mass loss and structure disintegration after 40 days. However, in the in vivo studies, the electrical output decreased rapidly within 3 weeks of implantation due to the possible swelling of the PLGA encapsulation coating. The applicability of such biodegradable biocompatible devices was further tested for providing electrical stimulation. The observation of neuron cell alignment directed by the electric field (10 V·mm^−1^) generated from the TENG device could be considered for neural repair applications wherein the device can be degraded and resorbed in the body without any adverse long-term effects. The approach was further extended in a later work by Li et al. [78], wherein the in vivo degradation process of such TENGs was controlled by employing Au nanorods, which responded to the near-infrared (NIR) light. The implanted TENG comprising of Au- Poly (1,8-octanediol-co-citric acid)—hemispherical array against PLGA worked well for more than 28 days, producing a ~2 V output. However, upon the application of NIR, the output rapidly reduced to 0 within 24 h and the device degraded within 14 days. When the in vivo output voltage was applied to fibroblast cells, it demonstrated a significant acceleration for cell migration which can be utilized for wound healing applications.

In their work, Lin et al. [72] have reported on the development of a self-powered heart-rate monitoring Body Sensor Network (BSN) system (see Figure 4). The single-electrode downy-structure TENG (D-TENG) powering the BSN utilized PTFE against copper and consisted of two basic units: (i) one segmentally alternating unit composed of copper back-coated PTFE thin films and copper thin films, which are stationary layers with one end being anchored onto the acrylic frame, leaving the other end free-standing, and (ii) a second unit of acrylic sheets segmentally adhered on the front and back surface by the copper-coated PTFE and copper thin films as the freestanding triboelectric layers, which are finally connected to the outer frame with a stretchable rubber. The structure is extremely sensitive to the external mechanical excitation and under the body movement of the wearer, the relative sliding of the triboelectric layer is maximized leading to the generation of power. An optimal D-TENG consisting of four grids was shown to produce 540 *V_p-p_* and a short-circuit current of 15 µA, providing an overall power of 2 mW (at 80 MΩ) at an excitation frequency of 10 Hz (overall efficiency of 57.9%). When worn on the arm, such a D-TENG was shown to power up the heart-rate BSN comprising of integrated heart rate sensor, power management circuit, signal processing unit, and a Bluetooth data transmission unit (displaying the heart-rate signal in real-time on a mobile phone).

A simple self-powered ultrasensitive pulse sensor (SUPS) based on nanostructured contact surfaces of Kapton and Cu (enclosed in PDMS) was proposed by Ouyang et al. [79]. The SUPS device when worn over the radial arteria generates a clear output voltage pulse of 1.5 V, 5.4 nA current with a high peak signal-noise ratio (45 dB), long-term performance (10^7^ cycles), and low price (<$1). By integrating the SUPS sensor with Bluetooth technology, a 16-bit A/D converter, and data storage capability, an end-to-end wireless pulse sensor device was developed. Even though the quality of the pulse signal is highly dependent on the sensor–skin interface, the SUPS was shown to capture the pulse signal from the carotid artery, the brachial artery, the radial artery, the finger, and the ankle artery region. Further measurements on live subjects were carried out to determine heart rate variability (HRV) and Poincaré plot of eight healthy subjects, six subjects with coronary heart disease (CHD), three subjects with atrial fibrillation (AF), and three subjects with atrial septal defect (ASD). The HRV Poincaré plot showed clear differences between these subject groups wherein the Poincaré plot of healthy subjects had an elliptical shape; while the plot of CHD patients had a shorter length/width than that of a healthy patient, which reduced further for an ASD patient, while the plot of an AF patient was relatively irregular. The SUPS was shown to provide an indicative diagnosis by measuring the pulse wave velocity (PWV) via the use of simultaneous use of two SUPSs to indicate the degree of arteriosclerosis. Further work (2019) from the same group has led to the development of a symbiotic pacemaker, a fully self-powered, implantable stimulator that utilizes TENG technology [80]. The fully implantable TENG achieved energy harvesting (open-circuit voltage of 65.2 V, 0.5 µA current), storage (external capacitor) as well as cardiac pacing on a large-animal subject by correcting sinus arrhythmia and preventing deterioration. This was enabled by the energy harvested from each cardiac cycle of ~0.495 μJ, which is higher than the pacing threshold energy of test-subject pigs (0.262 μJ) and humans (0.377 μJ). Additionally, the iTENG showed remarkable mechanical durability (100 million mechanical stimuli cycles) and cytocompatibility, which are typical determinants for long-term implantable devices.

**Figure 5:**
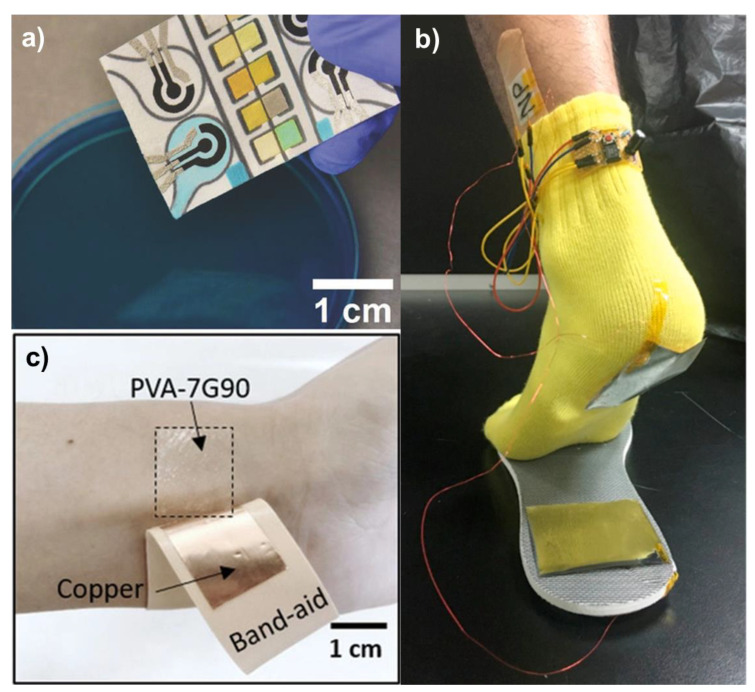
Example of wearable sensors, and where sample collection has been streamlined for simplicity. (**a**) Here the TENG-powered electrochemical sensor is dipped into the analyte solution and automatically imbibes the liquid, avoiding the requirement for pipettes, figure adapted from Pal et al. [21]. (**b**) Here a self-powered sensor is combined onto a bandage which can provide a comfortable, wearable experience for sensing lactate in-situ, figure adapted from Chen et al. [56]. (**c**) Here a wearable PVA film is used to power the sensing of cardiovascular information, figure adapted from Wang et al. [82].

Utilizing the excellent biocompatible properties of silk nanoribbons, Niu et al. [81] developed a fully biodegradable TENG comprising of silk nanoribbons and regenerative silk fibroin film with magnesium electrodes with an output performance of 41.64 V, 0.5 μA, and power density of 86.7 mW·m^−2^. The high sensitivity of such a TENG allowed it to be applied as a self-powered pulse sensor. Similarly, Wang et al. [82] developed an optimal mixture of PVA and gelatin to produce high dielectric constant films with large interfacial polarization which were then utilized for self-powered wearable cardiovascular monitoring (see Figure 5). As the pulse exerts a pressure less than 0.6 N·cm^−2^, its measurement requires a low detection limit and high sensitivity. When utilized for such measurements, the real-time triboelectric signal showed three distinct characteristic peaks corresponding to the human pulse, representing blood ejection, blood reflection from the lower body, and blood reflection from the closed aortic valve, respectively. Utilizing the time delay between the peaks, the authors were further able to calculate the radial diastolic augmentation index, which is a predictor of arterial stiffness.

In all the examples discussed above, a particular aspect of TENGs as wearable energy harvesters and sensors are brought forth—the requirement for a relatively large area (~10 to 25 cm^2^) which either needs to be worn/attached to the body or requires an invasive procedure. To overcome this, epidermal electronic systems—a new class of highly conformable, thin, and stretchable electronics, have emerged as an alternative approach to address the limitations of wearable biomedical devices [83]. The skin-like sensing interface conforms to the movements of the user while then matching the skin-impedance to provide a route for continuous monitoring of the metabolites such as glucose, lactate, and electrolytes like sodium, chlorine, and potassium in sweat, and has enabled real-time monitoring of wound status and autonomous drug delivery. Peng et al. [84] developed an all-nanofiber, E-skin-based TENG by electrospinning PLGA and PVA, with silver nanowires as electrodes. The selection of PVA and PLGA allowed the tuning of the antibacterial and biodegradable capability of E-skins which were further used to achieve real-time and self-powered monitoring of complete body physiological signal measurements. Sadri et al. [83], have reported on the development of self-powered electrophysiological bio-signal capturing for monitoring of ECGs, electromyograms (EMG), and electrooculograms (EOG) using epidermal triboelectric devices. The aptly named epidermal, nano texturized, triboelectric devices (EnTDs) use a triboelectric pair of ethoxy basis ethyl cellulose (EC) against a polyamide-6 backed up on thin Cu and Al foils. Following an extensive fabrication process involving soft lithography, laser cutting, and subsequent deposition of hydrophobic PDMS coating, the EnTDs were obtained which could be directly attached to the skin of the user. When the EnTD is stretched or bent, the relative sliding motion at the triboelectric interface of fluorinated EC nanograting and the polyamide surface leads to charge generation which can be modulated by varying the size and period of the nanograting. It should be noted that to account for the electrophysiological measurements, the three-electrode measurements need to be subtracted from the triboelectric signals which require a real-time baseline removal process requiring a separate microcontroller and thus represents a certain level of complexity when compared to other devices.

**Fulfilling REASSURED criteria:** Utilizing biocompatible Environmentally friendly materials, the implantable TENGs showed Robust behavior surviving over 100,000,000 mechanical stimuli cycles while providing enough energy to power pacemaker devices, meeting the REASSURED criteria. The in vivo implanted devices provide a clear pathway to develop Equipment-free devices.

#### 2.2.2. Respiratory Monitoring

Simple monitoring of human respiration characteristics (frequency 0.2–0.4 Hz, 12–16 breaths per minute at rest), respiratory flow, volume (~5 L·min^−1^), and specific oxygen (spO_2_) provides insight into the pulmonary health and physical condition of an individual [54]. In 2014, Bai et al. [85] reported on a membrane-based triboelectric sensor (M-TES) as a self-powered approach for measuring air pressure change for sensing, surveillance, and health monitoring. Utilizing a triboelectric pair of nanostructured-FEP against latex, the M-TES with dimensions of 3.7 cm × 3.7 cm × 2 cm, was able to resolve pressure changes of 0.34 Pa and 0.16 Pa, when the air pressure increased and decreased, respectively. Owing to its membrane-based structure, upon changes in the air pressure, the swelling latex membrane could contact-separate from the FEP forming an arc-shaped air cavity in the middle. When secured to the abdomen, the M-TES was able to measure the breathing rate (albeit slightly higher) of 32 breaths per minute in real-time. Similarly, Yi et al. [86] developed a stretchable rubber-based TENG which when wrapped around the patient’s chest was able to detect respiratory values. Utilizing the triboelectric pair of rubber (30 mm × 55 mm × 200 µm) against an anodized aluminum film (40 mm × 50 mm × 50 µm), the stretchable rubber-based TENG’s application as a self-powered breathing sensor was enabled by insulating the aluminum electrode from the body using a PTFE film (thickness: 50 µm). Under diaphragmatic breathing, voltages of up to 6 V were detected by the device, which could potentially be used for diagnosis of disease, medical treatment, and post-operative care. Chen et al. [87] reported on the development of a flexible self-powered blood oxygen monitoring system. Utilizing a specially designed ultrathin light-emitting diode (LED) and photodetector on Kapton films (1 cm^2^, 400 µm thick), the inherently flexible nano/microstructured PDMS and pre-stretched crumpled Au triboelectric pair provided high conformability to the skin. The TENG device was shown to provide a high voltage, current, and power output of 75.3 V, 7.4 μA, and 0.2 mW·cm^−2^, respectively, however, the test conditions were not fully specified. When mounted onto the thumb of a subject, the light emitted from the LED passes through the epidermis and is received by the photodetector after reflection, scattering, and deflection, containing information about the hemoglobin levels in the blood. Powered by the TENG, the LED-photodetector assembly was shown to detect a stable PPG signal which can be used to calculate the oxyhemoglobin saturation and pulse rate.

The TENG technology and applications have since developed significantly and the recent work by Jiang’s group showed a wireless respiratory-driven device to measure several respiratory parameters including ammonia (NH_3_) detection, as well as respiratory flow and frequency [88]. The monitoring of NH_3_ content in the patients’ breath is important as it represents the presence of uremic fetor which is often associated with chronic kidney disease. Wang et al. [88,89] utilized a Ce-doped ZnO-PANI nanocomposite film as a multi-functional layer performing the roles of the NH_3_-sensors sensing layer, electrode, and triboelectric surface in the TENG. To detect the respiratory pressure, a high-elasticity balloon was utilized to generate the pressure on the second triboelectric surface of the PDMS layer thus providing the contact-separation cycles between PDMS/Ce-doped ZnO PANI, corresponding to human respiration. At a constant respiratory flow of 5 L·min^−1^, a voltage of ~0.8 *V_p-p_* was obtained which was then utilized to track respiratory frequency, respiratory flow, and trace-level NH_3_ concentrations in exhaled breath. It was reported that the output voltage of the self-powered NH_3_ sensor monotonously decreased with the increase of NH_3_ concentrations from 0.1 to 25 ppm. When tested on a person with mouth ulcers, the TENG’s voltage dropped by nearly 21% which was ascribed to the presence of NH_3_ in the breath of the subject. In 2018, He et al. [90] developed a cellulose-fiber-based anti-bacterial TENG which can provide multiple functions of energy harvesting, particulate matter (PM2.5) removal, and respiration monitoring. The TENG consisted of biodegradable cellulose nanofibers (CNFs) deposited inside porous cellulose microfibers (CMFs) to create a nanostructured CMFs/CNFs paper. This was used against micro-punched FEP with Ag nanofibers as the back electrode as well as antibacterial surface. When implanted inside a surgical mask, the device produced a typical voltage of ~ 2.2 V at normal respiration and ~3.4 V after a short period of running and achieved a high efficiency of 98.83% of PM2.5 removal.

More recently. Kim et al. [91] reported on the development of a 3-D printed triboelectric respiration sensor (TRS) which can distinguish between human expiration and inspiration by chemically analyzing the carbon dioxide (CO_2_) concentration in respiration. To achieve this, a 10 wt.% polyethyleneimine (PEI) was dip-coated on one side of the aluminum electrode while an FEP thin film constituted the second triboelectric layer. Under the variable movement of the FEP film during breathing, a difference in the signal polarity was observed corresponding to the contact-separation cycle and was utilized to distinguish the inspiration and expiration conditions. When CO_2_ and nitrogen (N_2_) gases were injected in the Al/PEI-FEP 3-D printed TRS TENG, the voltage outputs were reduced by 7.6% and 11.7%, respectively, which was significantly higher than the control experiment without the PEI layer, where this reduction was ~1% only. The analysis of the output voltage allowed TRS to identify the four types of respirations: strong, weak, long, and short. However, the display of the device required an external Arduino which was not self-powered. Meng et al. [92] developed a woven self-powered pressure sensor for pulse wave and cuffless blood pressure measurements. Utilizing ITO as an electrode with interwoven PTFE and PET electrification layers, it was further encapsulated in PDMS for protection. The completed device exhibited a high sensitivity of 45.7 mV·Pa^−1^, a response time of ~5 ms, and high stability measured over 40,000 continuous operating cycles. An optimized low power consumption sensor system was further developed for monitoring pulse waves from the fingertip, wrist, ear, and ankles which transmitted the heart rate to a mobile phone via a Bluetooth connection.

Abnormalities in respiratory activities during sleep, including obstructive sleep apnea syndrome (OSAS), obstructive sleep apnea-hypopnea syndrome (OSAHS), and circadian rhythm disorder, affect nearly a billion people worldwide [93,94]. Nocturnal polysomnography—a standard clinical technique—is often used to monitor the condition of patients where monitoring of heart, lung, and brain activity, breathing patterns, arm and leg movements, and blood oxygen levels is carried out during sleep. However, this inherently complex method is intrusive, invades privacy, and requires the participation of a medically trained professional, which constrains its use in home healthcare and continuous monitoring [93,94]. Consequently, it is imperative to develop non-invasive, comfortable, cost-effective, and continuous sleep monitoring systems, some examples of which are discussed below.

OSAS is associated with the restriction and stoppage of the oral/nasal airflow for at least 10 s and may occur multiple times during sleep, presenting a significant immediate and long-term (coronary disease, cardiac ischemia, myocardial infarction, congestive heart failure, stroke, and nocturnal death) danger to the sufferer. Thus, regular monitoring of respiratory state during sleep is of high importance. Traditionally carried out using masks and ventilation pipes that are intrusive, the technology has since developed into wearable breathing sensors which are, however, still bulky and require an external power source [93,94,95]. Utilizing commercial nylon (30 µm thickness) and PTFE films (100 µm thickness) on copper foils (50 µm thickness), Zhang et al. [96] reported on a waist-wearable wireless sensor for sleep monitoring. Both thoracic and abdominal respirations were measured on two volunteers in real-time to show the Z-shaped TENG devices’ applicability. It was observed that the output voltage signal direction was opposite for thoracic and abdominal respiration, which was ascribed to the variation in abdominal circumference for these modes. The waist-wearable TENG device exhibited steady variation (constant frequency and peak-valley value of ~10–12 *V_p-p_*) of the breathing rhythms of the wearer, while a relatively long time of no voltage signal between two breathing rhythms provides a warning for the apnea. However, it should be noted that the signals transmitted to the mobile phones did not capture the true amplitude and were only representative of the respiration status. As the breathing rhythm’s low frequency (~ 0.7 Hz) is much lower than the optimal driven frequency range for energy harvesting by a TENG (usually 1–10 Hz), this led to a low instantaneous power of 0.23 μW at 300 MΩ load resistance. As the TENG could not fully power the wireless transmission system, an external power source in the form of a battery was required to enable the system, and thus the device could not fulfill the self-powered criteria [96].

Wang et al. [97] have utilized an airflow-driven single-electrode TENG for self-powered real-time respiratory monitoring by utilizing a n-PTFE film against a copper electrode in a constricted acrylic device. Under a nominal airflow volume of 120 L·min^−1^, the porous n-PTFE film (with a thin copper back electrode) provided low density for enabling the air flow-driven oscillations and, when contacted against copper electrode (on the surface of the acrylic), produced a voltage signal of ~4 V and 1 µA current. The real-time output of the TENG, recorded under different behaviors of slow, rapid, shallow, and deep breathing provided distinct corresponding respiratory signals. Additionally, the electrical signals produced by the n-PTFE TENG were used to quantify the volume of airflow during each breath cycle and such information is vital for patients with respiratory diseases like asthma and emphysema. A wireless respiratory monitoring and alert system was developed which utilized the TENG signal to trigger a wireless alarm in response to a change in the breathing behavior.

While the wearable TENG devices can provide continuous monitoring, their lack of flexibility owing to the presence of metal electrodes and acrylic backing, along with poor/little air-permeability, can affect the comfort of the subject. To overcome these challenges and to ensure a continuous contact between the electrode and triboelectric friction material, fiber- and textile-based TENG sensors have been envisaged. Zhao et al. [98] reported direct weaving of Cu-coated PET (Cu-PET) warp yarns and PI-coated Cu-PET (PI–Cu-PET) weft yarns on an industrial weaving loom. The triboelectric charges were produced at the crossing points of the weft and warp yarns and displayed a maximum voltage of 9 *V_p-p_* and a short circuit current density of ~15.5 mA·m^−2^. By integrating into a chest strap, the PI-Cu-PET fibers could monitor human respiratory rate and depth (amount of air which is effortlessly inhaled or exhaled in one breathing cycle) and could distinguish between deep, shallow, rapid, and slow breathing states. It should be noted that optimal signal processing techniques were utilized to remove false signals arising from muscle/chest movement as well as from the surrounding environment. The typical adult breathing frequency is of the order of 0.4 Hz and a 256-order finite impulse response low-pass filter with a cut-off of 2 Hz was utilized. Besides the excellent respiratory monitoring characteristics, the devices high air-permeability, lightweight, and flexibility make them highly amenable in the development of wearable applications. Finally, the fabricated TENG showed remarkable washing durability, in its ability to withstand standard machine-washing tests and did not affect its output performance.

Qiu et al. [99] reported on the development of a calibration-free self-powered pneumological relevant sensor for vital signs monitoring. A TENG based on electrospinning of materials utilized PANI as the electrode with polycaprolactone and thermoplastic urethane as the supporting layer on which PVDF and PA-6 were deposited as tribo-negative and tribo-positive materials. A device of 10 × 10 cm^2^ was able to provide a 1000 *V_p-p_* and 200 μA of current under a frequency of 2.5 Hz, and able to simultaneously power about 1000 LEDs. More importantly, owing to the excellent softness and gas permeability of the assembled TENG, the device could be used as a wearable smart health monitor where breathing could be monitored in real-time by distinguishing between normal, deep, and rapid breathing. Similarly, Meng et al. [71] reported a textile-based TENG which was utilized for personalized healthcare monitoring of pulse wave for OSAHS. The disease is characterized by narrowing of the upper respiratory tract, leading to an inability to maintain respiratory patency, and cycles of hypopnea and apnea during OSAHS are reflected in the heartbeat (consequently pulse wave) as they occur. The device utilized a silver-coated polyester fabric as the first triboelectric/electrode pair on which a flower-shaped design was stitched using a three-ply twisted polyester-metal hybrid fiber to form the second triboelectric/electrode layer. The textile TENG device employed two working principles: pulsating movement-induced fiber deformation and deformation-induced generation of electrical signals and was thus able to show 3.88 V·kPa^−1^ sensitivity for ambient pressure detection (range 0.1–4.3 kPa) and high stability for more than 80,000 cycles. The textile TENGs were attached directly on various clothing areas including headband, wristband, and shirt, to monitor the pulse waves at the forehead, wrist, and chest, respectively and the obtained voltage measurements mimicked the general pulse wave. By measuring the pulse wave amplitude (PWA) and pulse period (PP), the authors were able to distinguish the respiratory events associated with OSAHS in test subjects. Further integration into a wireless monitoring system, consisting of an A/D converter, microcontroller, and a Bluetooth module (with a corresponding mobile phone app) was shown as the case for an end-to-end system.

Zhou et al. [100] have reported on a multifunctional single-layered, smart textile for real-time continuous sleep monitoring. The functional fibers for TENG were constructed by wrapping an inner core of twisted conductive yarn with an outer sheath of ultra-thin silicone fibers which provided high flexibility when woven into a regular polyester bedsheet. These functional fibers provided a high sensitivity, broad working frequency bandwidth, high durability, and decent washability of 10.79 mV·Pa^−1^, 0–40 Hz, 20,000 cycles, and 8 washes for 20 min each, respectively, for each sensing unit. To monitor sleep, 60 sensing units of 10 × 13 cm were put together on an entire bedsheet in a matrix form. To monitor for physiological conditions, a 7 × 52 cm unit was utilized. It was reported that sleeping posture and superimposed vital signs of heart rate and respiration could be acquired from the smart textile. The heartbeat was quantified using a ballistocardiograph which clearly showed the presence of pre-systolic, systolic, and diastolic components. A small signal of ~0.5 *V_p-p_* which was produced due to regular breathing was distinctly absent in the case of an OSAHS episode.

Considering the examples discussed above for self-powered respiration and cardiovascular monitoring, it is abundantly clear that TENG technology provides a platform to develop sensitive, high-performance, and potentially conformable device solutions to monitor the conditions discussed above. It is expected that with the ever-growing aging population, the prevalence of these cardiovascular and respiratory diseases will increase, leading to a significant burden on the health systems. The development of smart, wearable sensors offering local data display as well as wireless transmission capabilities offers opportunities for healthcare professionals to remotely monitor the condition of patients and progression (if any) of the disease [101]. The extraction of other valuable data including heart rate, breath analysis, respiratory volume, depth, etc. using digital signal processing principles provides unprecedented access and insight into several health parameters providing a holistic view of the patient’s health. In the next section, self-powered sensors, and systems for the measurement of other biomedical and physical signals are discussed.

**Fulfilling REASSURED criteria:** The textile-based TENGs offer User-friendly and Equipment-free options for measurement of physiological signals and eliminates the need for further body-worn sensors or patient-side equipment.

#### 2.2.3. Other Self-Powered Sensors

Self-powered monitoring of small-muscle movement has been achieved in the literature wherein the contraction and relaxation of eye muscles drive an electrical signal from the TENGs directly placed on them. In the work reported by Anaya et al. [102], a unique topology of Non-Attached Electrode-Dielectric Triboelectric Sensor (NEDTS) was utilized to develop the Orbicularis Oculi muscle motion sensor for monitoring voluntary and involuntary eye blinks. The NEDTS is a unique topology in which the conductive electrodes are not bonded to the contacting dielectric surfaces, instead, due to the triboelectric interaction between the two elements in motion, voltage is generated in a separate conductor by non-contact electrostatic induction, allowing for near-field remote sensing. Relying on interaction between the human skin, the silicone-based material Ecoflex™, and a conductive polymer PEDOT, PSS-based films are used. The 1.5 cm × 0.6 cm × 2 mm NEDTS device attached near to the eye produced ~1.5 *V_p-p_*. The sensor motion was further amplified and digitized by a microcontroller to act as an interface to control a human–machine interface to assist people with mobility impairment.

Further work by Anaya et al. [103] has shown the development of a portable TENG-based sensor to monitor the movement of forearm muscles and tendons for people suffering from Parkinson’s disease. When the hand or fingers extend or flex, the gap between the dielectric polymer (Ecoflex™, 4 × 3.5 cm) and the conductor (Aluminum, 2 × 3 cm) deposited on a PCB changes, generating a voltage due to triboelectric contact. The device was used to assess localized bradykinesia and rigidity of fingers and hands, as well as wrist and arm tremors for each level of classification from “normal” to “moderate” status, as described in the Movement Disorder Society-Sponsored Revision of the Unified Parkinson’s Disease Rating Scale (MDS-UPDRS) examination of hand movement, finger tapping, and pronation-supination movement. Huang et al. [104] have shown the development of a dual-mode TENG combining the vertical contact-separation and single electrode aspects for self-powered human motion sensor. Their system comprised of Aluminium against nanostructured-polyethylene with specimen sizes for energy harvesting and motion sensor of 40 × 40 mm and 30 × 5 mm, respectively. At 90° bending angle of the index finger, a device of dimension of 30 × 5 × 2 mm exhibited a maximum voltage of about 320 mV while under identical bending conditions, the little finger shows a minimum voltage value of 110 mV, the middle finger and the ring finger show 230 and 180 mV, respectively. By changing different bending angles of the index finger, the detected voltage signals indicate that the increment of the index finger bending angle leads to an increase in the voltage signal which was attributed to a change in the contact area between the PE film and the Al electrode in the vertical contact-separation mode and the change in the capacitive coupling between the human skin and the device electrode in the single-electrode mode [104]. It is expected that the use of ultrasonication-based techniques for the synthesis of polymeric nanowires could enhance the voltage output, making the self-powered monitoring of joint movement much more sensitive [105]. Further work by the same group has demonstrated the development of soft, self-healing 3-TENG based to detect human motion [106] and more recently 4-D printed shape memory polymer based TENGs [107]. The self-healing polymer-based networks themselves were crosslinked by two kinds of dynamic bonds (imine bond and UPy). Poly(propylene glycol) bis(2-aminopropyl ether) (PEA) or bis(3-aminopropyl) terminated poly(dimethylsiloxane) (H_2_N-PDMS-NH_2_) was selected as soft segment for reaction with 2(6-isocyanatohexylaminocarbonylamino)-6-methyl-4(1H) pyrimidinone (UPy-NCO) and 1, 1, 1-tris[(4-formylphenoxy) methyl]ethane (FPME) to obtain flexible and healable polyazomethine (IU-PAM) and poly(dimethylsiloxane) (IU-PDMS) elastomers based on imine and UPy units [106]. Such a TENG could be used as a self-powered finger-joint motion sensor. For example, when the bending angle periodically changing from 30° to 60° and then to 90°, the output *Voc* was 0.6, 1.4, and 2.7 V, respectively. As the bending angle increased, the contact area of two triboelectric layers was enlarged, leading to rising the *Voc* of the TENG sensor simultaneously. Both the triboelectric layer and electrode could be autonomously self-healable under ambient environments using the heat produced from the human body providing the pathway to self-healing, wearable devices.

Underactive bladder syndrome (UAB), caused by the dysfunction of the detrusor muscle (myogenic UAB) or disease of the afferent system (neurogenic UAB), is defined as a prolonged or failed emptying of the bladder within a normal period, resulting in bladder dysfunction [108,109]. Current treatments of repeated multiple urethral catheterizations are highly invasive which may lead to urinary tract infections and asymptomatic bacteriuria. To avoid repeated invasive and/or surgical procedures, self-powered actuators and implantable force actuators have been proposed to empty the bladder [108]. Hassani et al. reported on the integration of these implantable actuators with a TENG-based sensor to detect bladder fullness for managing and treating neurogenic UAB [108]. The sensor incorporating shape memory alloy (NiTi, transition temperature 45 °C) on a PVC sheet, consisted of a deionized water-filled sponge layer and a PDMS layer stacked between Cu electrodes. When the applied force on the sensor was increased from 0 to 6.8 N, its output increased from 35.6 mV to 114 mV which corresponded to the volumetric change in the bladder. When the bladder was empty, the water remained in the sponge, thus minimizing the output triboelectric voltage, while upon the filling of the bladder, the deionized water was squeezed out of the sponge exposing the PDMS layer and leading to an increasing voltage output which leveled off when the bladder was full, actuating the sensor. As the actuator voided (emptied) the bladder, the sponge reabsorbed the water and the output voltage decreased back to its minimum value. The sensing mechanism allowed the TENG to indirectly measure the bladder fluid volume and when actuated, a voiding percentage of up to 78% of bladder volume could be achieved in an anesthetized rat.

Utilizing the same principles, mechano-neuromodulation of autonomic pelvic nerves was demonstrated using a triboelectric neurostimulator integrated with a flexible neural clip interface to control the bladder function [110]. Long-term validity of the neural clip interface was performed on bladder pelvic nerves in rats for 16 days to demonstrate the self-powered mechano-neuromodulation for bladder function in the future. Self-powered neuromodulation techniques were also utilized by Yao et al. [110] for in vivo vagus nerve stimulation for regulating food intake and obesity treatment. Comprising of a biocompatible flexible TENG attached to the surface of the stomach, the device generates biphasic electric pulses in response to the peristalsis (contraction and distention) of the stomach which can then stimulate the vagal afferent fibers to reduce food intake and achieve weight control. The device comprising of PTFE and metal layers was encapsulated in PDMS and Ecoflex™ for flexibility, stability, and biocompatibility, directly connected the TENG electrodes to the anterior and posterior vagus nerves of a rat using Au leads. Upon stomach contraction and distension, the triboelectric forward and reverse voltage and current (maximum output power of ~40 μW at 20 MΩ) were generated, which sent a cyclic alternating electrical signal to stimulate the vagus nerve as the stomach continued peristalsis. In their work, the strategy was shown to be successful on rat models wherein the average body weight was controlled at 350 g; 38% less than the control groups. The work, correlating nerve stimulation with targeted organ functionality through a smart, self-responsive system provides concepts for therapeutic technology using artificial nerve signals generated from body activities.

To overcome the limitations of the brittle piezoelectric ceramic-based transduction, self-powered load sensors in orthopedic implants have been explored. Ibrahim et al. [111] have reported on the development of a low-resolution load sensor for possible post-surgery total knee replacement (TKR) monitoring applications. The vertical contact-separation energy-harvesting TENG consisting of micropatterned Al and PDMS layers, separated by rubber springs, is for installation between the ultra-high-molecular-weight-polyethylene (UHMWP) bearing insert and the tibial tray. Under the application of a cyclical axial force (2.3 kN at 1 Hz), corresponding to the load-bearing capacity of the knee, a linear correlation between the root mean square output voltage (18 V_rms_ at an optimal load of 58 MΩ, average power 6 μW) and in vitro applied load was observed, thus confirming the device’s ability to act as a low-resolution load sensor. While never tested in an in vivo environment, the application of such TENG sensors could be useful in post-surgery evaluation and monitoring of load-bearing joints. Considering the examples of such implantable TENGs in/on internal organs, further exploratory research needs to be carried out for direct integration into implantable medical devices.

Guo et al. designed an FEP-Au based TENG to harvest mechanical energy from body movements to drive a commercial temperature sensor [112]. A typical 5 × 5 array of rhombic-shaped units, each of 3.6 × 2 cm, was shown to achieve ~220 *V_p-p_* and 460 nC of charge per cycle. When integrated into a larger 8 × 8 unit, the TENG was able to charge a paper-based supercapacitor of ~1 mF capacity to ~1 V within 250 s (impact frequency 3 Hz). Such a supercapacitor system was then utilized to run a temperature sensor requiring ~1.45 V to work which was powered by hand-flapping of the FEP-Au TENG. More recently, Zhang et al. [113] have developed wearable triboelectric-effect-based socks for scavenging low-frequency energy from natural human body movement to power a Bluetooth module and transmit the body temperature value detected by the embedded temperature sensor via an Internet of Things (IoT) framework. The textile-based contact-separation TENG comprised of four functional layers: a nitrile thin film and silicone rubber film with patterned frustum structures (acting as triboelectric layers), and two conductive textiles attached to the back of the two contact electrification layers for charge collection. The device was further sealed using two non-conductive textile layers. An output power of 0.32 mW (measured on a 44.4 MΩ load) was generated from 1 Hz walking, which was increased nearly 10 times in the case of running (2 Hz) with a maximum power of 3.18 mW (measured on a 21.3 MΩ load). Within ~300 times of normal walking steps, the output from the TENG sock was shown to charge a 27 μF capacitor to up to 8 V to power up a Bluetooth module to send the body temperature data to a smartphone. It should be noted that while some reports [114] have demonstrated TENGs comprising of temperature-sensitive materials to act as self-powered temperature sensors, given their poor resolution, accuracy, and sensitivity, it is anticipated that TENGs for such body temperature measurements would be utilized as a power source only and not as a sensor.

## 3. TENGs as a Platform Technology for Realizing REASSURED Solutions

**Figure 6:**
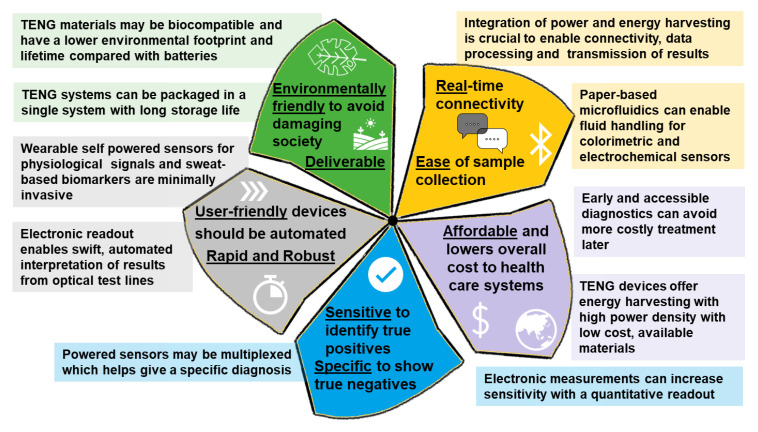
An overview of how Triboelectric Effect Enabled Self-Powered, Point-of-Care Diagnostics can be used to help achieve the REASSURED criteria.

As can be observed from the plethora of examples provided above, TENGs provide a favorable solution for developing a range of sensors and self-powered solutions. Among the many advantages of TENGs (compact, low-cost, disposable, etc.), the self-powered nature of the solutions offered by these devices makes them a favorable option in the development of REASSURED PoC solutions (Figure 6). While it can be argued that the ASSURED/REASSURED criteria focus on PoC diagnostics and not necessarily physiological monitoring, there is an obvious need for standardization of the physiological measurements, too. For rapid tests (such as SPED’s [21]) where the power for a brief period is required, TENGs are ideally suited, even if they fall short for continuous monitoring which is usually required for physiological monitoring solutions. Table 2 summarizes the different REASSURED principles and how existing TENG devices and complete TENG-based solutions contribute to these criteria.

As can be observed from the above table, the current state of the art, except for a couple of examples, cannot be yet regarded as a complete end-to-end, continuous, self-powered health monitoring system as the devices are not yet able to harvest enough energy to support the energy-intensive data acquisition and wireless transmission capabilities. As such, achieving long operation capabilities remains a grand challenge.

The authors believe that TENG-based end-to-end systems comprising materials that are abundant, low-cost, environmentally-friendly with low manufacturing costs offer the best chance for fulfilling the self-powered REASSURED criteria. For such analytical devices, paper will remain the material of choice owing to its accessibility, affordability, and ease of disposal compared to traditional materials used in microfluidics [116]. The capillary wicking properties of cellulosic paper can help drive fluid flow without an external pump which can be further tuned by modifying the paper’s chemical and surface properties. However, it is known that paper-based devices when exposed to ambient humidity get contaminated which may alter their flow characteristics and thus require high-quality sealing, which also helps enhance the flow rates as compared to open devices [117]. To this effect, da Silva et al. [117] have reported on the triboelectric effect induced sealing and flow rate control in microfluidic paper-based analytical devices using triboelectric effect.

**Figure 7:**
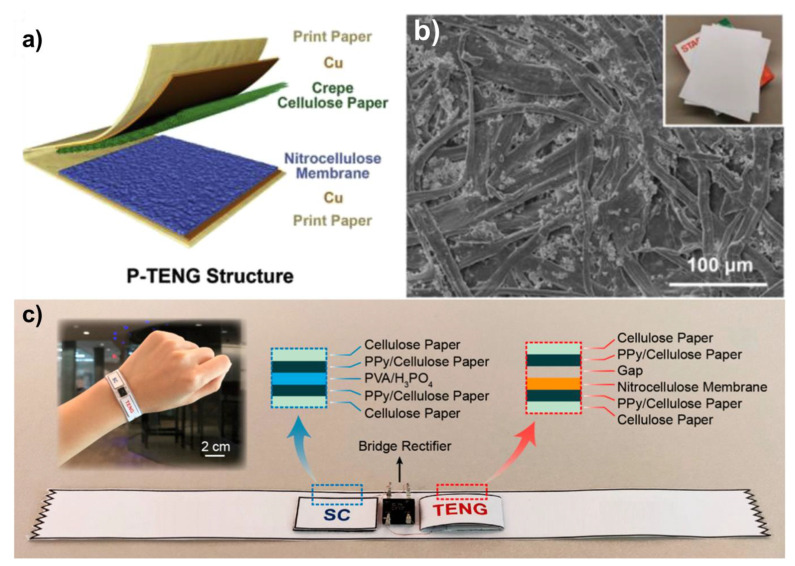
Example of self-powered paper-based devices. (**a**) Crepe cellulose and nitrocellulose membrane papers are used as environmentally friendly materials for triboelectric power generation, (**b**) SEM image of the cellulose structure is also shown, figure adapted from Chen et al. [119]. (**c**) Here, paper is used both for the power generation, with a paper-based TENG device, but also for storage using a paper-based supercapacitor, image adapted from Shi et al. [121].

At the same time, while not covered in the examples above due to the absence of complete self-powered monitoring devices, paper-based TENGs can offer a reasonable power density. For example, when utilized as a triboelectric surface, the effective dielectric modulation of cellulosic paper by BaTiO_3_ was shown to enhance the triboelectric output against a PDMS surface. The BaTiO_3_ dielectric modulated TENG demonstrates an output voltage of 88 V and a current of 8.3 µA, corresponding to an output power of 141 µW and an overall power density of 352.5 mW·m^−2^ [118]. Similarly, laser-induced graphene (LIG) based paper-polyimide TENGs showed significantly higher electrical output characteristics with a peak-to-peak voltage of up to ~625 V, a current density of ~20 mA·m^−2^, and a transferred charge density of ~138 μC·m^−2^ with a maximum power output of ~2.25 W·m^−2^, respectively, which was nearly 150% higher than the conventional aluminum electrodes [42]. The mechanically robust LIG electrodes show excellent stability with less than 5.0% variation in output over 12,000 contact cycles making them suitable for developing robust devices. A TENG utilizing a combination of crepe cellulose paper and nitrocellulose membrane exhibited excellent triboelectric performance with an output voltage and current of 196.8 V and 31.5 μA, respectively, with a high-power density of 16.1 W·m^−2^ at a load resistance of 1 MΩ [119]. Furthermore, paper-based substrates can enable the development of on-board energy storage devices such as flexible supercapacitors [120]. Cellulose paper with its porous bulk structure and rough and absorptive surface properties enables the construction of paper-based supercapacitors with a reasonably good performance for low-power electronics [120]. In recent work, Shi et al. [121] developed a polypyrrole-cellulose vs. nitrocellulose membrane TENG with a power density of 0.83 W·m^−2^ which was connected to a flexible solid-state paper-based supercapacitor comprising of PVA/H_3_PO_4_ gel as an electrolyte. The supercapacitor (of 90.1 mF·cm^−2^ capacity) itself utilized polypyrrole-cellulose as the electrode which when connected via a bridge rectifier, a self-charging power system could be obtained that was capable of harvesting and storing energy from the ambient environment (see Figure 7).

## 4. Challenges and Future Outlook

The presented review has highlighted and summarized the current state of the art of a wide TENG technology-based wearable and implantable sensors, and end-to-end systems for healthcare monitoring and PoC diagnostic applications. The TENGs were shown to not only act as self-powered sensors for high sensitivity and efficient measurement of bio-physiological/-medical signals but were also shown to act as sustainable power sources for driving complete measurement, processing, and display systems. The TENGs can therefore provide an efficient platform technology for the diagnostics and PoC industry owing to its high-efficiency, cost-effectiveness, facile materials and processes, and function-correlated output performance [54]. The alignment of these developments with the REASSURED principles for newly developed PoC diagnostics focusing on LMICs has also been summarized (Table 2).

Powering of various PoC device components (including automated sensing and readout, along with data communication) is key in the development of successful solutions, particularly in LMICs where electricity and other resources cannot be relied on. Various self-powered solutions exist, with this review focusing on TENG devices for providing power for PoC solutions. TENG devices offer several advantages towards REASSURED solutions, including low-cost, environmentally friendly, and manufacturability aspects, and may be integrated into functional PoC systems. Although these developments show promise towards achieving REASSURED solutions, there are several limitations to address and improvements to be made in the development of TENG-based PoC testing and monitoring devices, as described below.

### 4.1. Enhancement of Power Output of TENGs

Considering the examples discussed in the previous sections, there is a significant body of work that has shown the possible detection of vital signals and physiological monitoring through TENG-based sensors and the use of TENGs as energy-harvesting self-powered technologies, albeit the energy harvested was limited. For the TENG systems, the increase in the output power is usually achieved via either: (i) a judicious selection of triboelectric materials [46], or (ii) surface micro-/nano-structuring [122], surface chemistry modification [123], or (iii) charge injection [47] or composites with high-polarization piezoelectric additives [48] or finally (iv) physical hybridization of multiple energy harvesting technologies [124]. Except for the judicious use of tribo-materials, the other methods are reliant on expensive equipment and complicated processing steps including lithography, which defeats the low-cost advantage of TENGs and may not provide the required stability and reliability. Consequently, the selection of the optimal materials with high electron affinity difference is the simplest and most effective way to improve the performance of TENGs but is currently restricted by the limited choices of existing materials and thus demands the development of high charge exchange density materials, devices and architecture.

At the same time, while it is understood that the complete self-powered healthcare technologies are still some time away, the standardization of the devices’ energy harvesting capabilities and their output is required. Throughout our literature review, it was apparent that there are several instances where the data being provided is incomplete wherein it is sometimes missing output electrical parameters of either short circuit current, device impedance, maximum power output at impedance matched conditions, efficiency, or device dimensions, or sometimes even the testing conditions. The standardization and specification of all these devices and input/output conditions will help provide the readers as well as industry with information to correctly establish the current state of the art and thus the path going forward.

### 4.2. Efficient Power Management and Communication Circuits

While there is a significant amount of literature on the development of TENG materials and devices, system-level integration of TENGs in wearable devices and their practical demonstrations are not fully developed and are underexplored. Underpinning all the measurements and transmission is the power management circuit, which is responsible for rectifying, storing, coordinating, and is where required trickle charging processes for any devices take place. During the operation of the TENG where it would capture energy from the ambient environment or any human activities and the conversion of such AC power, there exist multiple pathways for losses and dissipation including DC conversion, transmission, and storage stages. Typical overall efficiency values of ~85% have been reported, suggesting that at least 15% of the energy is lost due to possible switching/conduction losses in rectifiers (requiring 500–700 mV for turn-on), MOSFETs, inductors, capacitors, and other circuit components. As the power generated by TENGs is usually of the mW order, to generate a useable DC voltage from the harvested energy, the design of a voltage-boosting rectifier is important [125].

As discussed in Section 1.2, the data processing (MCUs), storage (memory), and transmission (Wi-Fi, Bluetooth) blocks of the system architecture are the most power-hungry elements and provides the largest obstacle towards self-powered, end-to-end solutions. This necessitates a design trade-off/optimization between the communication and the onboard computation capability and thus requires an optimization of the data packet size and duty-cycling of the communication devices.

The abovementioned requirements are important considerations for any self-powering solutions for PoC diagnostic and monitoring applications. Specifically, for TENG-based solutions, integration with other components and scale-up needs to be considered as the PoC system moves through the development cycle. Limitations in terms of available power from TENG devices could be addressed by low-power electronic components in the most power-hungry parts of the system, or by simplifying the onboard electronics and computation required, as discussed above. The move towards fully REASSURED PoC testing and monitoring solutions cannot be fully accomplished by one component of the system; rather all elements need to integrate seamlessly to achieve the REASSURED criteria. TENGs provide a favorable powering platform towards this goal, but the surrounding components for sensing, processing, and communication need to be optimized to enable truly effective REASSURED solutions to be realized.

### 4.3. Data and Feature Extraction from Noisy Physiological/Biomedical Signals

For body-worn or any other sensor type which is continually monitoring a patient, a significant amount of data is being generated which requires the collection, storage, transmission, and more importantly analysis to correlate it to actionable values for informing clinical practice. The large datasets being generated call for meticulous extraction of multiple parameters using feature extraction and machine learning algorithms which themselves would be power-hungry and might require specific low-power consumption DSP processors. It is envisaged that the increasing uptake of physiological signal monitoring, the application of big-data analytical methodologies, and machine learning is inevitable.

### 4.4. Choice of Biofluid, Biomarkers, and Multiplexing

At present, many of the wearable self-powered electrochemical sensors, and particularly those using E-skin technology, primarily use sweat as a biofluid. Wearable self-powered platforms satisfy the REASSURED requirement for real-time, easy sample collection and being user-friendly. For example, Song et al. [115] have recently reported on the development of a self-powered wearable system consisting of a wearable freestanding-mode TENG, low-power wireless sensor circuitry, and a microfluidic sweat sensor patch on a single flexible printed circuit board platform to dynamically monitor and quantify key sweat biomarkers (e.g., pH and Na^+^). Similarly, colorimetric responses in functionalized porous substrates on soft, skin-mounted sensors electronics can yield chemical information, such as the pH of sweat, and further enable simple quantitative assays [126]. By combining these two approaches and low-energy image classification algorithms developed by Pal et al. [21], future devices could be built leveraging the TENG-enabled high sensitivity colorimetric quantification.

Most of the current results focus on measuring electrolytes and metabolites such as sodium, potassium, glucose, and lactate which are all in measurable quantities in sweat and act as markers for conditions such as dehydration, wound healing, and diabetes. However, sweat is also a rich source of protein biomarkers; Csősz et al. [127] quantified 95 proteins present in sweat, whilst Raiszadeh et al. [128] measured the presence of 180 proteins, many of which could offer a unique fingerprint for a range of health conditions, such as schizophrenia. The future possibility of using this vast range of available sweat protein biomarkers, for real-time and non-invasive multiplex measurements is an exciting prospect and should be a future challenge for self-powered wearable sensors. To achieve progress, engineers and biochemists should work closely together to develop platforms for monitoring proteins in sweat and thereby widen the types of disease that can be monitored. Furthermore, real-time monitoring of the concentration of medications within the body is a key application for which self-powered sensors can play a key role [129].

We believe the aforementioned challenges therefore provide ample research opportunities to further cement the TENGs as the platform of choice for developing self-powered physiological, biomedical, and PoC monitoring devices that can fulfill the REASSURED principles.

## Figures and Tables

**Table 1:** The energy consumption of the key components in a wireless sensor system monitoring PPG, ECG, and temperature simultaneously. Table adapted from Yu et al. [32].

Device Stage/Function	Typical Items/Components	Power Consumption	Conditions
Sensing/Transduction	Consumer photoplethysmography (PPG) sensor comprising of Light Emitting Diode (LED): KP1608SURCK PD:TEMD5080 × 01	240 µW from LED	LED working at 2 V and 20 mA with a pulse duration of 60 μs for 100 Hz sampling rate
Signal acquisition and processing	AFE4900	220 µW	PPG receiver (Analog Front End (AFE) and Analog to Digital Convertor (ADC circuits) working at 2 V with the current of 1 uA/Hz for 100 Hz
Microcontroller Unit (MCU), Memory and Wireless BLE (Low Energy Bluetooth)	Nordic nRF52832 NAND Flash MT29F4G 01ADAGDSF	11.1 mW, disable BLE 5 μW for standby 16.5 mW for BLE 3 μW for Page read 30 μW for Page write	Nordic nRF52832 working at 3 V (64 MHz): 3.7 mA, disable Bluetooth 9.2 mA, enable Bluetooth (0 dBm) 1 Mb/s; 27.6 mW in total, (27.6–11.1) = 16.5 mW for BLE 1.9 μA at standby mode.
			NAND 3.3 V, 37 mA for Page read/write. Time for read: 25 μs; for write: 220–600 μs (250 μs used for estimation) Page size: 2048 Bytes

**Table 2:** REASSURED principles and how existing TENG solutions contribute to the fulfillment of these criteria.

REASSURED PoC Device Criteria	How TENGs Can Fulfill the Criteria	Typical References
Real-time connectivity	TENGs provide built-in power on the device to enable data connectivity and/or transmission of results from the device using Bluetooth/wireless communication protocols.	TENGs with Radio/Wireless communications: Zhao et al. [63], Niu et al. [73], Wang et al. [97]
		TENGs with Bluetooth communications: Meng et al. [71], Lin et al. [72], Ouyang et al. [79,80], Meng et al. [92], Zhang et al. [96], Zhang et al. [113], Song et al. [115]
Ease of sample collection and Environmentally friendly	Sample collection and preparation take place on the rapid test or LFA itself. The energy harvesting TENGs and any other components would not play a direct role in this REASSURED aspect. However, solutions in direct contact with the skin that measure parameters directly from sweat negate sample preparation requirements. Although TENGs add to the materials and components that are used on the device, thereby contributing to the overall waste generated, many of the materials used in TENG devices are environmentally friendly or biocompatible. This offers a favorable solution compared to batteries and other power solutions which would be far more harmful to the environment. For fully REASSURED devices to be realized, power is a crucial component, and TENGs provide a promising solution for this in terms of environmentally friendly aspects.	Ease of sample collection: Self-pipetting: Pal et al. [21] E-skin: He et al. [60], Zhao et al. [63], Peng et al. [84]
		Wearables for physiological sensing: Ouyang et al. [79], Wang et al. [82], Meng et al. [92], Zhang et al. [96], Qiu et al. [99], Zhang et al. [113], Song et al. [115]
		Biocompatible materials based TENGs: Ma et al. [66], Zheng et al. [74,75], Yao et al. [110], Liu et al. [76], Zheng et al. [77], Li et al. [78], Ouyang et al. [79,80], Wang et al. [81], Wang et al. [82], Peng et al. [84], Hassani et al. [108]
Sensitive and Specific (performance)	Sensitivity and Specificity can be defined collectively as the performance of the PoC test. TENGs provide power to sensing, processing, and readout modules which provide automated and sensitive readout from a test—much more so than manual readout by a user. Colorimetric sensing enables quantitive and sensitive distinction between colors and intensities to be made, compared with visual inspection by a user. Electrochemical detection, which can be powered by TENG devices, is highly sensitive and enables analyses that are not possible using manual user inspection techniques. Physiological signal monitoring powered by TENGs enables sensitive and continuous readout of signals for cardiovascular, respiratory, and other applications.	Electrochemical Sensing: Pal et al. [21], Chen et al. [56], Zhang et al. [59], Liu et al. [76], Chen et al. [56]
		Colorimetric detection: Pal et al. [21]
		Chemical detection: Chen et al. [56], He et al. [60], Wang et al. [88,89], Kim et al. [91]
		Physiological Monitoring: Meng et al. [71], Niu et al. [73], Ma et al. [66], Wang et al. [82], Peng et al. [84], Bai et al. [85], Chen et al. [87], Meng et al. [92], Zhang et al. [96], Wang et al. [97], Song et al. [115]
User friendly	TENG devices provide power and thus automation of various functionalities, including sensing and readout components on the device. This eliminates the need for user interaction and manual readout by a user, which can result in errors. For TENG-powered monitoring of physiological signals, automated and continuous readout can be achieved, with little to no user interaction required.	Qualitative display via LED switch-on: He et al. [60]
		Automated display of results: Pal et al. [21], Lin et al. [72], Meng et al. [92], Zhang et al. [96], Zhou et al. [93], Song et al. [115]
		Monitoring of physiological signals without user interaction: Meng et al. [71], Niu et al. [73], Ma et al. [66], Wang et al. [82], Peng et al. [84], Bai et al. [85], Chen et al. [87], Meng et al. [92], Zhang et al. [96], Wang et al. [97]
Rapid and Robust	TENGs provide power to detection modules which enable heightened sensitivity to be obtained, often resulting in faster test readout (e.g., optical sensors can detect finer colorimetric changes than the human eye, potentially detecting the result sooner). Capture and communication of the results are faster with an automated, connected PoC device where TENGs power on-board communication modules. Monitoring of physiological signals using TENG devices provides instantaneous data which can be used for rapid diagnosis. Robustness focuses on the storage conditions of the rapid test, for example, humidity and transport. However, the robustness of developed TENG components and the integration with detection, processing, and communication modules is an important consideration for device success. TENG devices rely on repetitive mechanical movements and are typically strong devices.	Rapid: 28 s for electrochemical testing of glucose, lactate, and uric acid: Pal et al. [21]
		Robust: ∼350,000 in-vivo contact-separation cycles: Ma et al. [66]
		80,000 loading-unloading cycles: Meng et al. [71]
		100,000,000 pulse sensing operation: Ouyang et al. [79]
		100,000,000 mechanical stimuli cycles for implanted iTENG: Ouyang et al. [80]
		40,000 continuous operation cycles: Meng et al. [92]
		20,000 sleep monitoring cycles: Zhou et al. [100]
		16 days in-vivo vagus nerve stimulation: Yao et al. [110]
Equipment-free	Although TENGs add additional components, they introduce additional functionality by providing power, which in turn drives sensors, electronics, readout, and communication components and eliminates the need for external equipment (e.g., power supplies or a laboratory)	Sample to answer glucose, lactate, and uric acid measurements with on-device LCD readout: Pal et al. [21]
		Heart-rate measurement with a mobile app readout: Lin et al. [72], Meng et al. [92]
		Respiration monitoring system with mobile app readout: Meng et al. [71], Zhang et al. [96], Wang et al. [97]
Deliverable to end-users	Although TENGs introduce additional components to an ASSURED device, which in turn also require manufacture and deployment, they assist towards the long-term goal of realizing a fully integrated PoC device solution. Once an integrated stand-alone solution is achieved—using TENGs to drive the on-board functionality—the solution could be deployed to end-users in a contained package and at an affordable price.	Cost: Paper and portable potentiostat based electrochemical devices: Pal et al. [21], (<$21)
		Pulse sensor for antidiastole of cardiovascular diseases: Ouyang et al. [79], (<$1)
